# Identifying a stochastic clock network with light entrainment for single cells of *Neurosporacrassa*

**DOI:** 10.1038/s41598-020-72213-1

**Published:** 2020-09-16

**Authors:** C. Caranica, A. Al-Omari, H.-B. Schüttler, J. Arnold

**Affiliations:** 1 Department of Statistics, University of Georgia, Athens, GA 30602 USA; 2 Department of Biomedical Systems and Informatics Engineering, Yarmouk University, Irbid, 21163 Jordan; 3 Department of Physics and Astronomy, University of Georgia, Athens, GA 30602 USA; 4 Genetics Department, University of Georgia, Athens, GA 30602 USA

**Keywords:** Systems biology, Cellular noise, Oscillators, Regulatory networks, Single-cell imaging, Stochastic modelling, Stochastic networks

## Abstract

Stochastic networks for the clock were identified by ensemble methods using genetic algorithms that captured the amplitude and period variation in single cell oscillators of *Neurospora* *crassa*. The genetic algorithms were at least an order of magnitude faster than ensemble methods using parallel tempering and appeared to provide a globally optimum solution from a random start in the initial guess of model parameters (i.e., rate constants and initial counts of molecules in a cell). The resulting goodness of fit ${x}^{2}$ was roughly halved versus solutions produced by ensemble methods using parallel tempering, and the resulting ${x}^{2}$ per data point was only ${\chi }^{2}/n$ = 2,708.05/953 = 2.84. The fitted model ensemble was robust to variation in proxies for “cell size”. The fitted neutral models without cellular communication between single cells isolated by microfluidics provided evidence for only *one* Stochastic Resonance at one common level of stochastic intracellular noise across days from 6 to 36 h of light/dark (L/D) or in a D/D experiment. When the light-driven phase synchronization was strong as measured by the Kuramoto (K), there was degradation in the single cell oscillations away from the stochastic resonance. The rate constants for the stochastic clock network are consistent with those determined on a macroscopic scale of 10^7^ cells.

## Introduction

One of the main challenges of systems biology is explaining the dynamic behavior of single cells with their stochastic intracellular variation^1,2^. This stochastic intracellular variation has profound consequences on the regulation and phenotypes of genetically identical individual cells^3,4^. One example is the effects of stochastic intracellular variation on the dynamics of genes and their products involved in the biological clock^5,6^. While populations of 10^7^ cells/ml display highly synchronized behavior producing regular oscillations at the macroscopic scale, the behavior of individual cells is quite different. There is now evidence that individual cells in *Neurospora* *crassa* have clocks^5^, but there is substantial variation in phase between the clocks in different cells. What mechanisms at the single cell level explain how cells oscillate, and how do these cells come to oscillate in phase on a macroscopic scale?

There are three hypotheses for how cells come to oscillate as they transition from the single cell level to the macroscopic level. One possibility is that there is some form of chemical signal shared between cells that allows cells with different clock phases to reinforce and synchronize each other^6,7^. A second possibility is that the noise itself can play a positive role in generating oscillations^8^, and the mechanism for noise producing oscillations can invoke a physical hypothesis for biological oscillators known as Stochastic Resonance^9^. A third possibility is that there is some cell cycle gated mechanism that imposes regular oscillations on single cells^10,11^.

These three mechanisms can be examined using flow focusing microfluidics^12^ to capture individual cells under particular conditions for observation and to manipulate the environment of the cell to test individually these hypotheses under the effects of a variety of factors, such as light^6^. The conditions of the experiment here are used to isolate and test the Stochastic Resonance Hypothesis. Single cells are isolated in different droplets for observation so that they cannot communicate. Also single cells are maintained in media so that they cannot divide^6^. In this way the effects of cell-to-cell communication and cell cycle-gating on the clock can be eliminated. Only the mechanism of Stochastic Resonance remains to be examined^9^.

**Figure 1:**
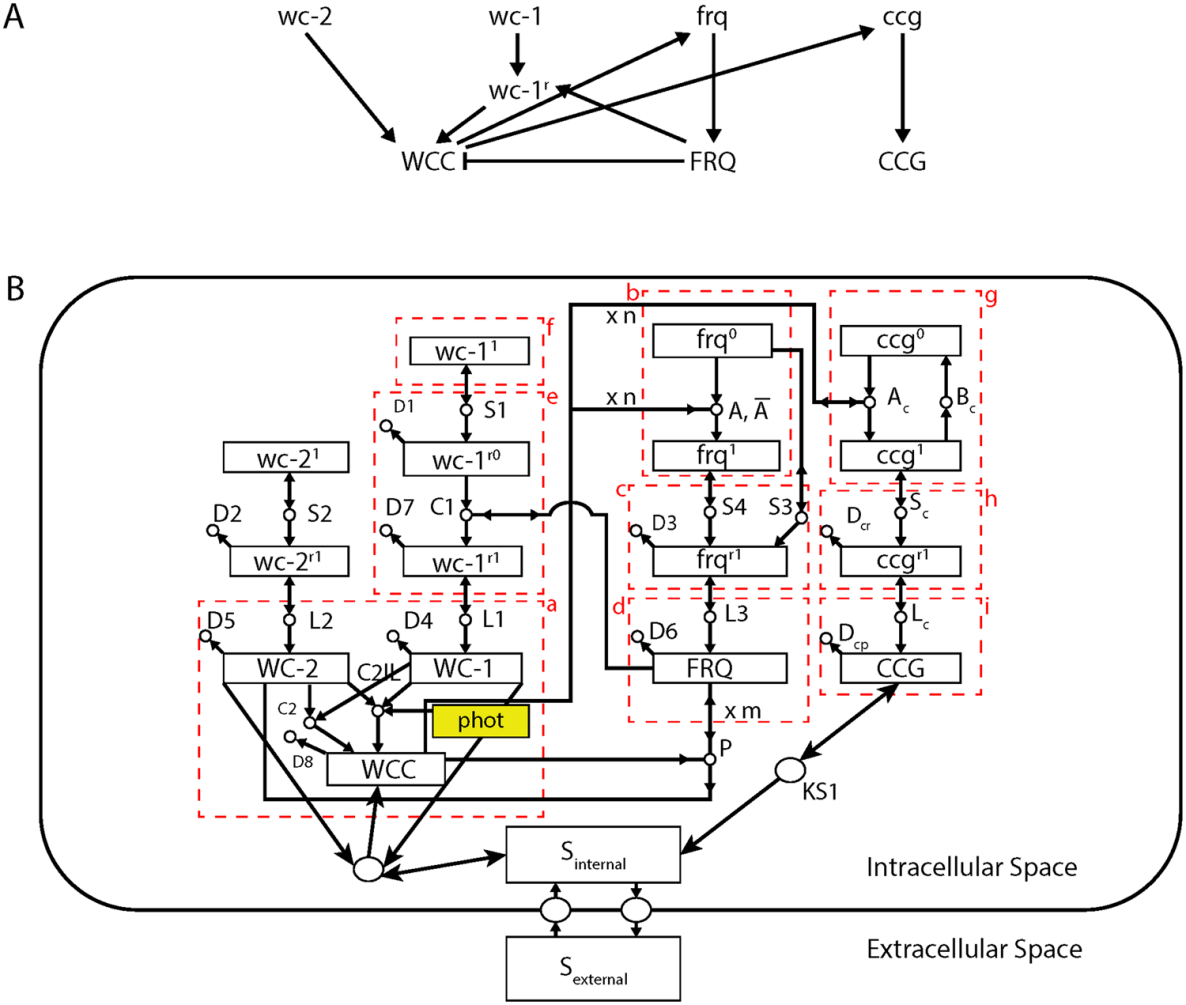
(**A**) The key elements of the clock stochastic network are summarized. There are both a negative feedback loop, in which WCC activates the gene *frq* encoding the oscillator protein and a positive feedback loop in which the FRQ protein stabilizes the *wc-1*^*r*^ mRNA. The genes *wc-1* and *wc-2* are the positive elements in the clock, while the *frq* gene is the negative element in the clock. (**B**) The full specification of the model is given by the network in panel (**B**). Circles denote reactions, and boxes represent reactants and products in the network. Double arrows denote catalytic reactions. The labels on reactions do double duty as both label for the reactions and as rate coefficient(s) for a particular reaction. Those reactions with no resultant product constitute decay reactions. All proteins and mRNAs have decay reactions as examples. The red dotted boxes denote components of the network across which there is approximately no net flow of molecules. Typically the dotted boxes are only crossed by catalytic reactions. Modified from^14^.

The Stochastic Resonance Hypothesis can be viewed as a prediction of a reasonable null hypothesis or “Neutral Model”^13^ specified by a stochastic clock network (Fig. 1) that does not invoke any other mechanism to explain clock-like behavior. A stochastic network has several elements: (1) the variables in the network are counts of molecules in a cell; (2) the counts of molecules or molecular species (typically on the order of 100–1000 s) are small^14^; (3) a network of chemical reactions connects the molecules (Fig. 1); (4) the reactions occur “randomly” over time^15^; (5) with each reaction’s occurrence the species involved are incremented or decremented. For example the reaction A + B—> A + C would decrement the count of B by 1 and increment the count of C by 1. Since the counts of molecular species are small within a cell, under the neutral model a major cause of change in the molecular species over time is the random drift in molecular counts due to stochastic intracellular noise within the cell^16,17^. Yet under certain circumstances this simple stochastic reaction network (Fig. 1) does predict that circadian oscillations will arise in populations of cells (i.e., Stochastic Resonance)^14^. Before invoking any more complicated hypothesis involving, for example, communication between cells to explain the emergence of oscillations in populations of cells, it is necessary to overturn this simpler model and its predictions.

These stochastic networks differ from the deterministic network models for the clock^18,19^ in that the molecular species are counts rather than concentrations^14^; moreover, the trajectories of molecular species are stochastic and unpredictable in contrast to deterministic models, in which once the initial molecular species concentrations are known along with the rate constants in each reaction (Fig. 1), the whole history is predictable. As the counts of molecular species are made large, then in the limit the deterministic models can sometimes be recovered^20^. These stochastic networks have one additional key parameter over deterministic models, the “size” of the cell, which determines the level of noise in the counts of molecular species. When single cell measurements are taken on a genetic network as here^5^, the simpler deterministic models are no longer appropriate (see, for example the single cell trajectories in Supplementary Fig. S0 from earlier work^6^).

In previous work the data on ~ 1591 isolated cells was used to test the adequacy of a stochastic clock network in the dark (D/D) to provide initial evidence for the Stochastic Resonance hypothesis^14^. Here we use additional light entrainment data on single cells to construct a stronger test of this neutral model for explaining clock-like properties and to explore its limitations using light entrainment of single cells under a 6 h, 12 h, and 36 h artificial day with equal amounts of light and dark (L/D)^5^. In addition to providing a stronger test of the neutral model the light entrainment data can also be used as a stronger test of the Stochastic Resonance Hypothesis.

Examining the neutral stochastic clock network without communication and without cell cycle gating is of particular interest under varying light regimes. In some models and experimental systems oscillators are hypothesized to have limits to their ability to entrain to an external entrainment signal—if the driving signal has a period sufficiently far from the intrinsic period of the cell oscillator, then entrainment fails^21^. These entrainment limits have been examined in the mammalian Suprachiasmatic Nuclei (SCN)^22^.

The model filamentous fungus, *N.* *crassa*, is particularly well suited to test this neutral model for the clock because isolated cells maintain circadian oscillations^6^. The *N.* *crassa* model system is then complementary to cells in the SCN, which cannot usually sustain oscillations when isolated^23^. The *N.* *crassa* system is also complementary to another major model system for the clock, the cyanobacterium, *Synechoccocus* *elongatus*, because *N.* *crassa* can entrain to light at the single cell level^5^. In contrast *S.* *elongatus* shuts down transcription in the dark, making it more difficult to study light entrainment^24^. Thus, *N.* *crassa* is particularly well suited to use both dark (D/D) and light entrainment experiments (L/D) to provide a strong test of the neutral hypothesis of the clock stochastic network and Stochastic Resonance.

There are several questions to be addressed about this neutral model: (1) is the stochastic network of the clock consistent with the available single cell data? (2) if not, how does the model fail? (3) Is there a limit to the neutral model’s ability to explain light entrainment data?^21^ (4) When the amount of single cell data is quadrupled to include light entrainment, how does support for the Stochastic Resonance Hypothesis hold up with light entrainment data and data in the dark?

**Figure 2:**
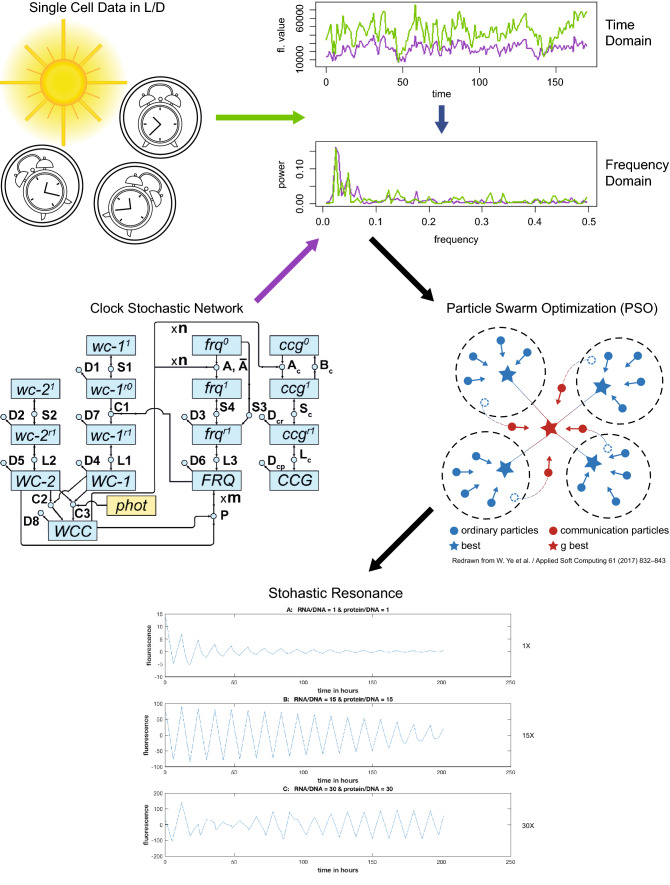
Single cell data are collected under a Light/Dark (L/D) regime and compared with predictions from a stochastic clock network. Trajectories of model (in purple) and experiment (in green) for the comparison are transformed into the frequency domain. Particle Swarm Optimization is used to fit the model to the single cell data in the frequency domain. The fitted model displays one stochastic resonance for one common level of noise and L/D and D/D regimes. This stochastic resonance displayed by the model is the hypothesis to be tested.

Since the manuscript is in some places quite technical, a road map for the manuscript is now provided as well as a graphical abstract (Fig. 2). The end point and message for this work is the last figure, demonstrating a stochastic resonance in the fitted network. Below or above the stochastic resonance the circadian rhythms of single cells are degraded. At the beginning the model is laid out. The novel element to this stochastic network for single cells of *N.* *crassa* is the inclusion of light as a molecular species. The structure of this network is suggested by earlier deterministic models on the macroscopic scale of 10^7^ cells/ml^18^. Then this stochastic network for single cells is fitted to the average periodogram or power spectrum using ensemble methods originally introduced by the authors to systems biology from statistical physics^25^. In the initial implementation of these ensemble methods for the D/D data it was found that the Metropolis–Hastings method of Markov Chain Monte Carlo (MCMC) was insufficient for identification of the model ensemble, but more sophisticated parallel tempering methods were successful in identifying the stochastic network for single cells in the dark (D/D)^14^. So, parallel tempering methods for fitting the clock stochastic network became the natural starting point for fitting model ensembles to L/D data here. These more complicated stochastic networks with a light response proved to be a challenge for parallel tempering methods. It was necessary to develop a novel approach to ensemble methods using genetic algorithms^26^. Genetic algorithms represent a very broad class of optimization methods^27^, and here two recently developed genetic algorithms^28,29^ were used to identify model ensembles.

Then an assessment of goodness of fit was made using the Hilbert Phase^30^, which is functionally independent of the period and amplitude captured in the average periodogram of single cells^31^. Consideration of the phase over time showed precisely where the model ensemble succeeded and where the ensemble needed improvement when the effects of light synchronization were weak. Stochastic networks have one additional parameter, the “size of a cell”, that determines the level of stochastic intracellular noise in a cell. An empirical approach was developed to identify the level of stochastic intracellular noise in a cell by relating the noise to the cell’s RNA/DNA and protein/DNA ratios. The model fitting was shown to be robust to variation in the RNA/DNA and protein/DNA ratios and hence in the level of stochastic intracellular noise. Having identified a promising “neutral model” with no other hypothesized factors affecting cellular phase synchronization, it was demonstrated that there was only one stochastic resonance in the fitted ensemble for a variety of L/D experiments and that as the noise was varied away from this stochastic resonance, circadian oscillations were degraded. These last two observations are the major points of the paper.

## Model

The neutral model for each genetically identical cell is a stochastic network displayed in Fig. 1B, and the broad outline of its features are given in Fig. 1A. The network of genes and their products begins with three clock mechanism genes in Fig. 1A: (1) the gene *frequency* (*frq*) encoding the oscillator protein FRQ; (2) one of two activator genes, *white-collar-1* (*wc-1*) encoding WC-1; and (3) the second of two activator genes, *white-collar-2* (*wc-2*) encoding WC-2. The positive elements WC-1 and WC-2 are transcription factors that form a White-Collar Complex (WCC)^32^. In Fig. 1A the WCC protein activates the oscillator gene *frq*, which in turn produces ultimately the FRQ protein, which is involved in deactivating the complex WCC. This negative feedback loop in part explains the origin of oscillations at the macroscopic scale^18^. There is also a positive feedback loop involving FRQ acting on the *wc-1* mRNA (*wc-1*^*r*^) in Fig. 1A. The “stabilization” of this *wc-1* mRNA by FRQ is crucial to explaining oscillations as well at the macroscopic scale^18^.

The details of how the stochastic network functions are given in Fig. 1B. A single cell is described by the counts of genes and their cognate messenger RNAs (mRNAs) and protein (boxes) in a cell. The molecular counts of species change at rates (the labels on circles) associated with the different reactions (circles) in the kinetic network. As examples, all clock mechanism genes (*frq*, *wc-1*, and *wc-2*) are transcribed at a rate Sx (e.g., S4) and translated at a rate Lx (e.g., L3). Messenger RNAs(mRNAs) and proteins decay at a rate Dx. The key reactions for oscillations at the macroscopic level are the rate of activation/deactivation (A and of $\stackrel{-}{A}$) of the oscillator gene *frq*, the rate of deactivation P of WCC by FRQ, and the decay rate D7 of the stabilized mRNA *wc-1*^*r1*^^18^. There is a total of 23 reaction rates and 12 initial conditions for a total of 35 parameters in this model.

Genes under the control of the clock mechanism are called *clock-controlled* *genes* (*ccg*). One *ccg* of particular interest is the hypothesized gene that produces the autoinducer or quorum sensing signal S_i_ synchronizing the clocks in different cells (indexed by i). Under the neutral hypothesis the rates of production of this signal are zero (i.e., if K_S1_ = 0). There is no communication hypothesized between cells in this paper in that cells are isolated in different droplets. Another is the recorder gene *ccg-2P*:mCherry in the MFNC9 strain for observing the operation of the clock (i.e., the hands on the face of the clock)^33^.

The one novel feature from earlier work^14^ is the presence of photons as a light species in Fig. 1B. This introduces novel light (C3) and dark reactions (C2) for the production of WCC as in earlier work^19^ (see Fig. 2 of this earlier work). This slight extension of the model can be shown to be formally equivalent to another network with only one reaction (${C}_{2}+ {C}_{2}{I}_{L}s\left(t\right)$) producing WCC that varies with time in the following way.

A photon species named phot is introduced in Fig. 1 whose temporal trajectory is not obtained from solving the Master Equation by the Gillespie Algorithm^34^ but is given to us. The concentration [phot] is an exogenous variable of the form:

$$\left[phot\right]={I}_{L}s\left(t\right),$$

where ${I}_{L}$ is the light intensity and $s(t)$ switches between “Light On” (L), with “On”-intensity I_L_, and “Light Off” (D), after every time interval t_LD_, starting with “L” at time t_L,0_:

$$\begin{gathered} {\text{s}}\left( {\text{t}} \right) = {\text{1 for t}}_{{{\text{L}},{\text{n}} - {1}}} \leq{\text{t}} < {\text{t}}_{{{\text{L}},{\text{n}}}} {\text{ and}} \quad {\text{n}} = {1},{3},{5},{7}, \ldots \, \left( {{\text{i}}.{\text{e}}.,{\text{ if n is odd}}} \right) \hfill \\ {\text{s}}\left( {\text{t}} \right) = 0{\text{ for t}}_{{{\text{L}},{\text{n}} - {1}}} \leq{\text{t}} < {\text{t}}_{{{\text{L}},{\text{n}}}} {\text{ and}} \quad {\text{n}}= {2},{4},{6},{8}, \ldots \, \left( {{\text{i}}.{\text{e}}.,{\text{ if n is even}}} \right). \hfill \\ {\text{t}}_{{{\text{L}},{\text{n}}}} = {\text{t}}_{{{\text{L}},0}} + {\text{ n t}}_{{{\text{LD}}}} \quad {\text{ for n}} = 0,{1},{2},{3},{4}, \ldots \, . \hfill \\ \end{gathered}$$

So, for the experiments in^5, 6^ as an example for the 12 h day, the specification of the switch s(t) would be:

$${\text{t}}_{{{\text{LD}}}} = {\text{ 6h}},\quad {\text{I}}_{{\text{L}}} = { 53}00{\text{ lux}}, \quad {\text{t}}_{{{\text{L}},0}} = \, 0{\text{ h}},$$

assuming t_L,0_ = 0 h is the time when the L/D exposure cycles were started.

There is then one rate for a L/D experiment of the form:

$${C}_{2}+ {C}_{2L}{I}_{L}s\left(t\right)$$

Since we cannot separate the product ${C}_{2L}{I}_{L}$, we treat it as one parameter called ${C}_{2IL}$ in Fig. 1B, which has the same units as ${C}_{2}$ and is defined as C_2IL_ = C_2_*f_IL._ The parameter ${C}_{2}$ is specified from the D/D experiments, and the parameter ${f}_{IL}$ affecting illumination was initially set to 2, but then was allowed to float in the fitting. The parameter ${f}_{IL}$ is initially taken to be constant across L/D experiments because these experiments were done on the same apparatus and conditions except for variation in the length of the day^5^.

The production of [WCC] under all light regimes can then be described by a single reaction, a reaction having the rate given by ${C}_{2}+ {C}_{2}{f}_{IL}s\left(t\right)$.

## Results

A graphical abstract for the whole workflow in the results section is given (Fig. 2).

### Single cell data used to identify the stochastic clock network

Single cell trajectories on CCG-2^33^ were obtained and made publicly available^5^ under a variety of light conditions for cells: (1) in the dark (D/D); (2) under a 6 h day (L/D); (3) under a 12 h day (L/D); (4) under a 36 h day (L/D) (see “Materials and methods”). The fluorescence of single cells isolated in droplets were captured every 1/2 h for ~ 10 days^5^. An example of the stochastic variation in trajectories in a D/D experiment is provided (Supplementary Fig. S0) from earlier work^6^. Over 94% of the variation in these trajectories is stochastic intracellular variation^6^. These trajectories were then Rhodamine B normalized and detrended with a moving average^35^ for constructing periodograms and Hilbert Phase for fitting to a stochastic network^5^.

### Obtaining the fitted stochastic network to the single cell data in both D/D and L/D entrainment experiments

Ideally the fitting process would use all of the data in the trajectories (Fig S0)^36–38^. The challenge is that the individual trajectories are quite noisy and out of phase with each other^6^. One might be tempted to use the average of these trajectories (in red in Supplementary Fig. S0), but the averaging just removes the periodic signal. Grima has suggested a fast and tractable fitting procedure based on a meaningful summary of the trajectories in Supplementary Fig. S0^39^. There are three features of a periodic process, its period, amplitude, and phase. The periodogram or “power spectrum” summarizes two out of three of these features. This statistic is what is used for fitting. First, the trajectories (Supplementary Fig. S0) are detrended, and then the periodogram is calculated for each single cell trajectory and averaged^6^. The result is a meaningful summary of the data because it captures the period locking of single cell trajectories reported in previous work^5^. Detection noise is removed from the periodogram (see “Materials and methods”). Then the models are fitted by Markov Chain Monte Carlo Methods (MCMC) to the average periodogram of cells on GPUs^14^ using the criterion in (2) in “Materials and methods”.

**Figure 3:**
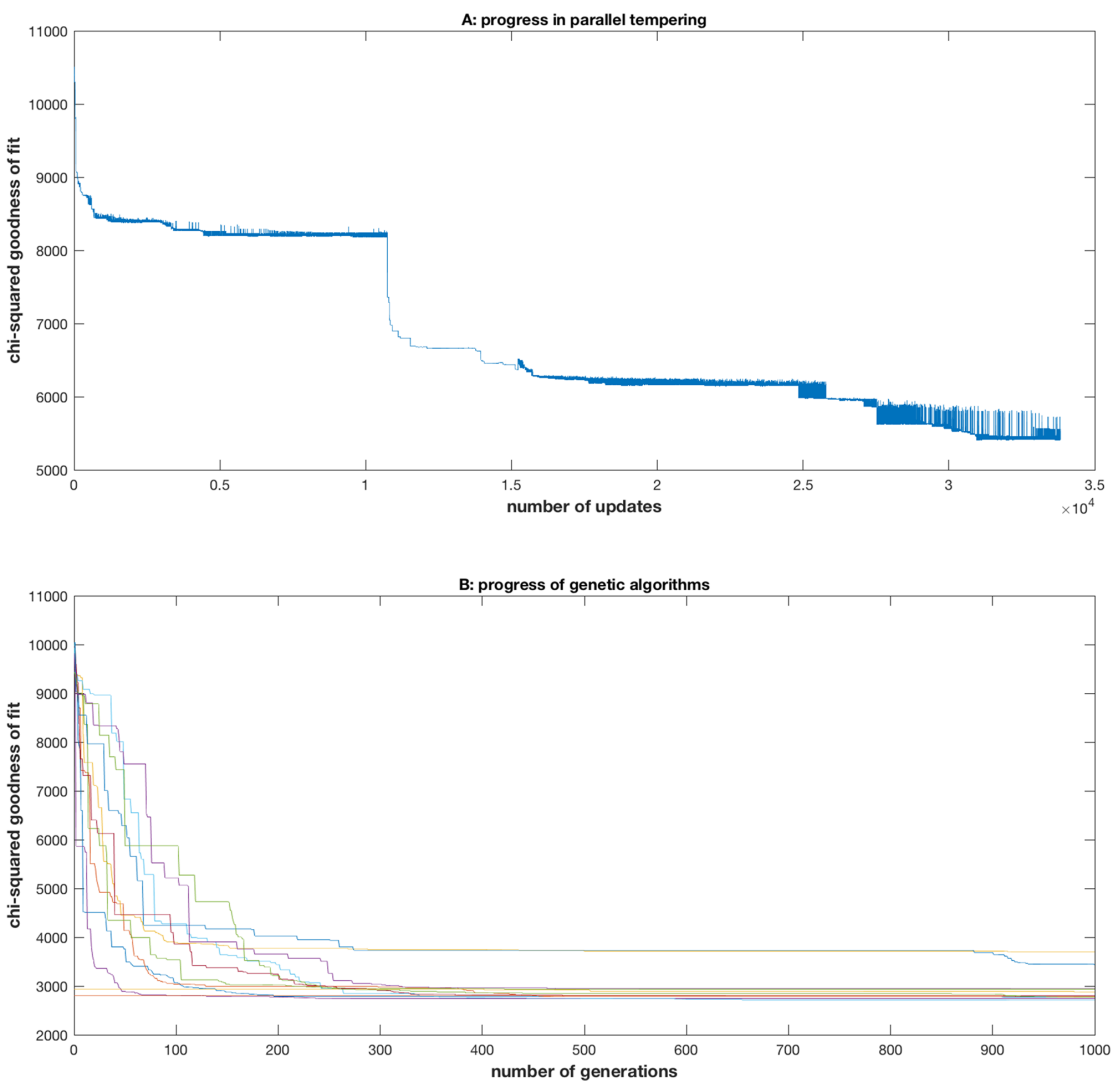
The chi-squared goodness of fit statistic improved during a Monte Carlo simulation used for fitting the model ensemble in Fig. 1 to average periodograms for the D/D experiment and 3 L/D experiments using parallel tempering (**A**) or genetic algorithms (B). In every case the genetic algorithms outperformed parallel tempering. (**A**) The first stage finished after 15,117 updates. The second stage finished after 25,684 updates. The first stage involved 17 chains; the last two stages involved 60 chains. The final third stage allowed the illumination parameter ${f}_{IL}$ to float as a parameter. (**B**) Twelve genetic algorithms in Table 1 converged to approximately the same solution from either a random start or Sobol space filling sequence in the 35-dimensional parameter space^40^. Two poorer performing genetic algorithms in Table 1 were set aside. The plots were created in MATLAB_R2018B (https://www.mathworks.com/products/matlab.html).

The equilibration process to fitting the ensemble occurred in three stages by parallel tempering (Fig. 3A) described in “Materials and methods”. In the first stage the grid of temperatures was allowed to grow to 17 chains with 15,117 updates. Beginning with an initial ${\chi }^{2}$ = 10,507, the ending achieved was ${\chi }^{2}$ = 6,371. In order to promote further communication between replicas at different temperatures, the temperature grid was expanded to 60 chains with 10,567 updates for a final ${\chi }^{2}$ = 5,977. In the final stage the illumination parameter ${f}_{IL}$ was allowed to float in the fitting process from a value of 2. In the final stage the fitting improved to a ${\chi }^{2}$ = 5,410 after 8,030 more updates with 60 chains as shown in Fig. 3A.

### Strong test of the Neutral Model with light entrainment data on single cells

In previous work the neutral model in Fig. 1 was tested against D/D single cell fluorescent data on the MFNC9 strain with a CCGp:mCherry recorder alone using a periodograms (of model and data) with 256 frequencies^14^. The power of the 21 h signal in the model could be varied by changing the amount of amplification occurring during transcription and translation in the network (Fig. 1B)^14^. With little amplification the final molecular counts in the cell of the CCGp:mCherry recorder would be smaller and noisier; with substantial amplification the final molecular counts would be larger and less noisy. In this way the strength of the circadian signal could be examined versus the stochastic intracellular noise inherent in a cell’s molecular counts. The model ensemble fitted to the D/D data alone predicted Stochastic Resonance in which the power in the periodogram spectrum associated with a 21 h peak varied nonlinearly with the level of stochastic intracellular noise^14^.

Here we constructed a much stronger test of the neutral model in Fig. 1 by introducing light entrainment data for days of varying length: 6 h day, 12 h day, and 36 h day. The amount of data used to identify the stochastic network was four times that in earlier work^14^. This model is neutral in the sense that there is no communication between cells because the cells are isolated in droplets^5^. We quadrupled the amount of single cell data used to fit the clock stochastic network in Fig. 1A to single cell data on four experiments, in which each experiment provided trajectories on over 1,000 cells every half hour for 10 days^5^. Together all four experiments produced 953 frequencies in the power spectrum for fitting the ensemble. The data are publicly available (see “Materials and methods”). The data can be thought of as protein levels on a CCG protein every half hour over 10 days in each experiment. One further piece of information is that relaxation experiments have been constructed previously on single cells to document that the response to light is not simply that of a driven system, but involves a self-sustained single cell oscillator with its own intrinsic period of 21 h responding to the L/D cycle imposed^5^.

Parallel tempering was used to fit the stochastic network in Fig. 1B to the experimental periodograms on the four experiments. In the accumulation run the final chi-squared statistic (see “Materials and methods”) was ${\chi }^{2}$ = 6,496.55 with n = 240 + 256 + 201 + 256 = 953 frequencies in the periodograms computed from the best of three independent Monte Carlo runs reaching finishing stage 1 (Fig. 3A). The number of unknown parameters in Fig. 1B was 34. The contribution of each data point in the periodogram was then ${\chi }^{2}/n$ =6.82. This fit was inadequate as detailed in the supplement, and a new approach was developed using genetic algorithms^27^.

The steps leading up to the successful use of genetic algorithms can be found in the supplement. In a genetic algorithm 20–80 particles (i.e., models) were created in 5, 10, or 20 swarms to live on the 35 dimensional parameter space (with the illumination parameter ${f}_{IL}$ being fitted as well)^28,29^. The swarms of particles moved stochastically in the parameter space as specified by equations (3–4) or (3–5) in “Materials and methods” during a generation. The best particle at the end of 600 or 1,000 generations (Table 1) was used to initiate a Metropolis–Hastings Monte Carlo accumulation run^14^. A distinct genetic algorithm was also tried in Table 1 with different dynamics in (3–5) and no recombination. The genetic algorithms were implemented on GPUs.

**Table 1:** Genetic algorithms with characteristics below were used to optimize the likelihood function in (2) and produce an ensemble of models.

Method	No. of swarms M	Particles per swarm N	Initialization of particles	Number of generations (iter)	Final ${\chi }^{2}$
DMS-PSO-CLS^29^	5	4	Sobol	1,000	4,607.65*
DMS-PSO-CLS	20	4	Sobol	1,000	2,773.07
DMS-PSO-CLS	10	4	Sobol	1,000	2,781.44
DMS-PSO-CLS	10	4	Random	1,000	3,703.9
DMS-PSO-CLS	10	4	Random	600	2,743.5
PSO-DLS^28^	10	4	Random	600	2,933.5
**PSO-DLS**	**5**	**4**	**Sobol**	**1,000**	**2,708.05**
PSO-DLS	20	4	Sobol	1,000	2,797.88
PSO-DLS	10	4	Sobol	1,000	3,436.22
PSO-DLS	10	4	Random	1,000	2,772.33
PSO-DLS	10	4	Random	1,000	2,880.11
PSO-DLS	10	4	Random	1,000	2,941.89
PSO-DLS	5	4	Random	1,000	6,702.86*
PSO-DLS	20	4	Random	1,000	2,768.15

Each run was initialized with $\theta$-parameters either initially positioned on a space-filling Sobol sequence^40^ or randomly within the 35-dimensional parameter space including the illumination parameter ${f}_{IL}$ (see “Materials and methods”). All genetic algorithms were run for 600–1,000 generations to equilibrate the search for an optimum to Eq. (2).

*These two algorithms had only 20 particles and were eliminated from further consideration.

A total of 14 such independent runs of the genetic algorithms was conducted with 5, 10, or 20 swarms, and 4 particles in a swarm to examine the impact of genetic algorithm and swarm number, for example, on calculation time and finding the optimum to (2). There was no significant difference in the final chi-squared statistics between the two types of genetic algorithms in Table 1 by a Wilcoxon Rank Sum Test at the 0.05^41^.

In the first run, the chi-squared statistic was reduced by almost half from the best parallel tempering run with ${\chi }^{2}$ = 5,410 in Fig. 3A to ${\chi }^{2}$ = 2,708.05 by the best genetic algorithm (in bold in Table 1). All genetic algorithms, using a random start on the parameter space, outperformed parallel tempering (Table 1). The chi-squared statistic per data point was then ${\chi }^{2}/n$ = 2,708.05/953 = 2.84, which is better than other published ensemble fits by deterministic models on the macroscopic scale^19^ as well as ensemble fits by stochastic models to the D/D data alone^14^. The longest time for an equilibration run with a genetic algorithm for an 80 particle swarm was 25 h. This is an order of magnitude faster than the equilibration run using parallel tempering in Fig. 3A. Two 20 particle algorithms were eliminated from the competition for poorer optimization results (Table 1), leaving 12 competing genetic algorithms.

**Figure 4:**
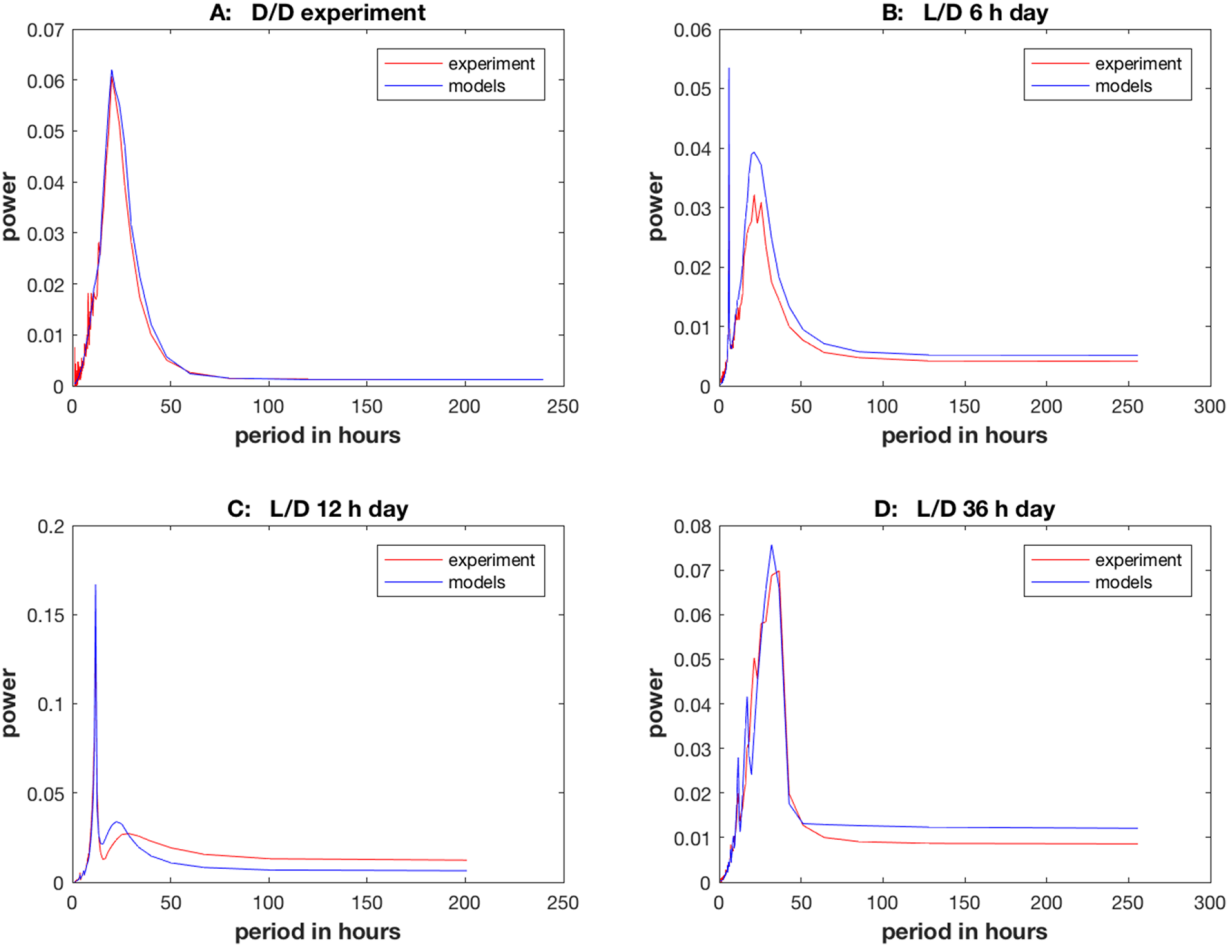
The average periodograms for single cells as a function of period for four experiments (D/D, L/D with 6 h day, L/D with 12 h day, and L/D with 36 h day) were fitted very well by the model ensemble (${\chi }^{2}=2708.05)$. (**A**) D/D experiment; (**B**) L/D with 6 h day; (**C**) L/D with 12 h day; (**D**) L/D with 36 h day. Models fitted were obtained by a genetic algorithm (using 10 swarms and 20 particles) described in “Materials and methods”, and data are the same as in Fig. 3, but power is presented as a function of period in each periodogram. The period is the inverse of the sampled frequency, namely $\frac{1}{{f}_{l}}, l=1,\dots ,\left[L/2\right]$. The plots were created in MATLAB_R2018B (https://www.mathworks.com/products/matlab.html).

To capture the behavior of the cellular clocks under the model ensemble derived from the best genetic algorithm, the four periodograms were plotted as a function of period (i.e., the inverse of the sample frequency ${f}_{l}$ ) in Fig. 4 rather than the index of the frequency as in Supplementary Fig. S1. As can be seen, the fit is extraordinarily good. For example, the model and experimental periodograms are hard to distinguish in Fig. 4B. In Fig. 4B,C the model tracked quite well to the 6 h and 12 period, respectively. The model succeeded completely in tracking to the period at the driving frequency of the light signal. Over the range of a 6 h day to 36 h day there was no observed limitation to the ability of the model to produce a population of oscillators that tracked to the day experienced, unlike the limit to entrainment for cells in the SCN^21^. In conclusion, the introduction of genetic algorithms appeared to support the hypothesis that the limits of entrainment seen in cell tracking in Supplementary Fig. S1 to the driving light signal, using parallel tempering, is an artifact of not finding the maximum to Eq. (2).

One further test was conducted using the remaining 12 independent runs of genetic algorithms to ascertain whether the optimum in Eq. (2) was local or global. As can be seen in Fig. 3B, all runs converged approximately to the same chi-squared statistic, strongly suggesting a global optimum of the ensemble had been achieved. Each of the 12 independent runs in Table 1 was then used to construct an accumulation run of 14,000 updates with Metropolis–Hastings Monte Carlo^14^ and combined to produce a final reconstruction of the likelihood in (2) together with its summary of the parameter distribution in Table 2 (as described in “Materials and methods”). The best model in the accumulation run had a ${\chi }^{2}$ = 2,671.95 (Supplementary Table S1).

**Table 2:** Ensemble means and standard errors indicate that the parameters in stochastic network for single cells are tightly specified by Markov Chain Monte Carlo using genetic algorithms with the D/D experiment and three L/D experiments.

Parameter	Initial Parameter values from MCMC Deterministic model ensemble (Yu et al.^18^)	Initial Parameter values from published ensemble (column 2) in molecular number units of stochastic network from D/D experiment^14^ (column 3)	Mean parameter values from model ensemble computed by Parallel tempering for D/D experiment	Standard error (SE) of parameter value across ensemble computed by parallel tempering for D/D experiment	Mean parameter values from model ensemble computed by genetic algorithms for D/D and L/D experiments in Metropolis–Hastings accumulation run	Standard error (SE) of parameter value across ensemble computed by genetic algorithms for D/D and L/D experiments in Metropolis–Hastings accumulation run
Number of cells	–	1591 (D/D only)	1591 (D/D only)	1591 (D/D only)	4 experiments (D/D + 3 L/D)	4 experiments (D/D + 3 L/D)
u_r0	3.99924	113	2,156.705728	68.14603254	249	1.94493283
u_r1	0.442441	18	22.46137677	0.872953544	266.5025	1.24742302
u_p	4.24E−07	459	2,144.149238	68.74768856	267.75	1.41999973
f_0	0.356365	1	0.465055176	0.01143672	033,333,333	0.00785783
f_1	0.0824576	0	0.534944824	0.01143672	0.6666667	0.00785783
f_r	4.90E−06	31	59.15869679	2.637452857	263.66667	1.53551295
f_p	3.0804	345	2,534.336311	77.72114325	258.83333	0.96610217
w	9.24126	101	55.40042039	1.674320488	280.11306	1.34419102
g_0	0.0066195	1	0.71623752	0.010337149	0.5	0.00833449
g_1	2.59E−06	0	0.28376248	0.010337149	0.5	0.00833449
g_r	1.17E−06	26	35.67840252	1.030258983	234.41667	1.45223506
g_p	1.37E−05	102	59.19075145	4.920903774	283.93833	1.01849186
A	0.000658482	6.06E−13	2.56E−10	7.31E−12	2.24E−10	1.10E−11
Abar	0.546986	0.546986	1.589532708	0.035661845	0.6046986	0.01326434
S1	0.061594783	83.70771546	80.12566921	0.302471515	82.372323	1.03259487
S3	0.00146575	3.569116497	0.400641074	0.036565894	13.491623	0.42722599
S4	2.2396	5,453.449297	8,316.020583	100.2852188	77.524423	1.13421469
D1	0.723678	0.723678	1.294999006	0.030289616	71.2760416	2.63473865
D3	0.299703	0.299703	4.382612039	0.181101578	6.16109147	0.17691415
C1	0.0428595	4.81E−05	0.000932789	2.47E−05	9.22E−05	4.25E−06
L1	31.7758	4.244678204	4.777735371	0.106626479	2.60684774	0.04948995
L3	3.02387	0.485087349	0.665600817	0.011127036	0.09315913	0.00269597
D4	0.00323262	0.00323262	0.08474029	0.004700587	0.05674687	0.00194278
D6	0.15183	0.15183	0.193685712	0.002236097	12.0326238	0.28052851
D7	0.138387	0.138387	2.130911791	0.090030385	0.11260178	0.00432734
D8	0.00248668	0.00248668	0.007744621	0.000182717	0.00014153	3.61E−06
C2	0.162687246	0.162687246	1.515554675	0.077548547	95.9668318	3.1203002
P	19.5648	3.12E−11	2.72E−09	4.83E−11	3.46E−08	6.82E−10
Ac	4.06813	7.82E−09	1.86E−08	2.55E−09	3.44E−05	1.89E−06
Bc	2.52197	2.52197	2.581096866	0.040197442	0.88230334	0.0199371
Sc	1.01E−06	73.80414613	61.51499414	1.109629713	11.0853255	0.25892314
Lc	1.15E−08	2.231095711	1.61524392	0.017335914	0.01161864	0.00012954
Dcr	0.219758	0.219758	0.150810052	0.00291715	0.27129774	0.00638981
Dcp	0.696903	0.696903	0.54063952	0.006141903	0.0224811	0.00035852
fIL			–	–	16.4900328	0.38777036

The parameters including the initial numbers of molecules and the rate constants in Fig. 1 are labeled in the first column. In the second column are the parameter values from a deterministic model ensemble^18^ on a macroscopic scale, in which WC-2 is constant over time. In the third column the parameters from the deterministic model ensemble are reported in units appropriate for the stochastic network as molecular counts. The last four columns are the ensemble means and standard errors (across the ensemble) generated by parallel tempering (see “Materials and methods”) for a single cell experiment with 1,591 single cells in a D/D experiment^14^ or from four experiments by genetic algorithms (D/D + 3 L/D experiments).

One standard control for MCMC experiments is to plot the parameter values in an accumulation run versus sweep (i.e., the time taken on average to visit once to each parameter in the model) (Supplementary Fig. S2). If the accumulation run were not complete, there would be trends in some parameters with sweep. All of the plots showed little trend, indicating that the accumulation run was successful. The plots also display which parameters are well specified in the ensemble. For example, the *wc-1* stabilized mRNA decay rate (D7) and the protein–protein interaction (C1) are tightly specified, while other parameters, such as the FRQ protein decay rate (D6), are not tightly specified. The horizontal lines in Fig S2 represent MCMC accumulation runs of different genetic algorithms in Table 1.

### What are the kinetic rules for the clock at the single cell level and how do they compare with the rules at the macroscopic level?

The series of 3 light entrainment experiments with the D/D experiment with 953 frequencies in the 4 periodograms in Fig. 4 provided a strong test of the clock network in a single cell, but they also provided precise estimates of the rate constants and initial gene and cognate mRNA and protein counts in a cell as well (see standard errors in last column of Table 2). These parameter estimates (i.e., rate constants and initial conditions for molecular counts of species) provided a means to determine if single cells play by the same or different rules than cells in aggregate at the macroscopic level^19^.

A comparison was made between a published ensemble only derived from the D/D data using an accumulation run from parallel tempering, in which an adequate fit was obtained^14^, with an ensemble (Table 2) derived from D/D data together with the L/D data using an accumulation run from genetic algorithms that also provided a remarkably good fit to the combined data set (Fig. 4). Parallel tempering required an informed initial guess to the parameters (column 2) from fitting the network on the macroscopic scale^18^. This initialization needed to be converted to molecular counts as described in “Materials and methods” (column 3) to provide an initial guess for parallel tempering. The results of parallel tempering are shown (columns 4 and 5). These “best practice” results are then compared with the results from genetic algorithms (columns 6 and 7). In comparing these two model ensembles (columns 4 vs. 6) there was remarkable agreement in the specification of the genetic network, but there are several changes in the rate constants from the estimates based only on the D/D experiment in Table 2. For example, the translation rates (L1, L3, and Lc) were lower on the clock mechanism genes based on the 4 experiments vs. the one D/D experiment with 1,591 cells.

The biggest surprise is in the mRNA stability of *wc-1*. In the fitting of the model to all 4 single cell experiments the derivative mRNA *wc-1*^*r1*^ was more stable as measured by the decay rate D7 than in the D/D single cell experiment alone. Having a stable *wc-1*^*r1*^ mRNA has been argued to be essential for oscillations at the macroscopic scale^18^. In the network fitted to all of the single cell data the modified *wc-1* mRNA, *wc-1*^*r1*^, is stable. For example, the decay rate D7 = 0.11 ± 0.0043 under all 4 experiments with a long lifetime of 1/D7 = 8.88 h as measured macroscopically^18^ versus D7 = 2.13 ± 0.09 in the D/D experiment alone. The single cell data in the D/D experiment alone was not sufficient to confirm this result found macroscopically. For models fitted to the D/D experiment alone, the decay rate (D7) was found to be D7 = 2.13 ± 0.09^14^. Evidence against the parallel tempering method being the cause of the discrepancy in the decay rate (D7) comes from the fact that fitted ensemble achieved by parallel tempering was an adequate fit to the average periodogram of the D/D data. As a caveat, if we had implemented a longer equilibration run with parallel tempering, we might have achieved the results of MCMC runs using genetic algorithms reported here. When the lines of different genetic algorithm accumulation runs are close together, as for the translation rate (Lc) for *ccg-2P:mCherry* (Fig S2), that is indicative that different MCMC runs converged to the same optimizing parameter value. For instance, in the case of the translation rate the ensemble covers the values from 0.002 to 0.02.

Also a comparison was made between the ensembles computed here using parallel tempering (Fig. 3) and with those using the genetic algorithms (Table 1) with respect to the illumination parameter ($${f}_{IL})$$ on a common data set (D/D + 3 L/D experiments). In allowing the illumination parameter to float, the final value of $${f}_{IL}$$ achieved a much larger value of 16.49 ± 0.39 than that derived under the use of parallel tempering, namely $${f}_{IL}$$= 2.

There are two sources of variation captured in the standard errors in Table 2 on these parameters. There is variation in the standard errors across models, and there is also variation in the parameters estimates due to stochastic intracellular noise. Both sources of error are reflected in the standard errors. In addition to the standard errors in Table 2, there are histograms of the rate constants (Supplementary Fig. S3). Some parameters, such as the decay rate of the stabilized *wc-1* mRNA (D7), are quite tightly specified, while other parameters such as the transcription rate of *frq* (S4), have considerable variation.

Generally in comparing the rate constants obtained from all four experiments (column 6) to those derived from macroscopic experiments (column 2)^18^ using Euclidean distance on the parameters in common, the agreement was much better than just based on the D/D experiment alone (column 4)^14^. The only rate constant out of line with the macroscopic limit appeared to be the decay rate D6 of FRQ^18^. There is also considerable variation in the estimates of this decay rate (Supplementary Fig. S3). The conclusion is single cells appear to play by similar rules as aggregates of 10^7^ cells.

### The stochastic intracellular noise level (i.e., the size of the cell) can be experimentally determined as a parameter in the model

**Figure 5:**
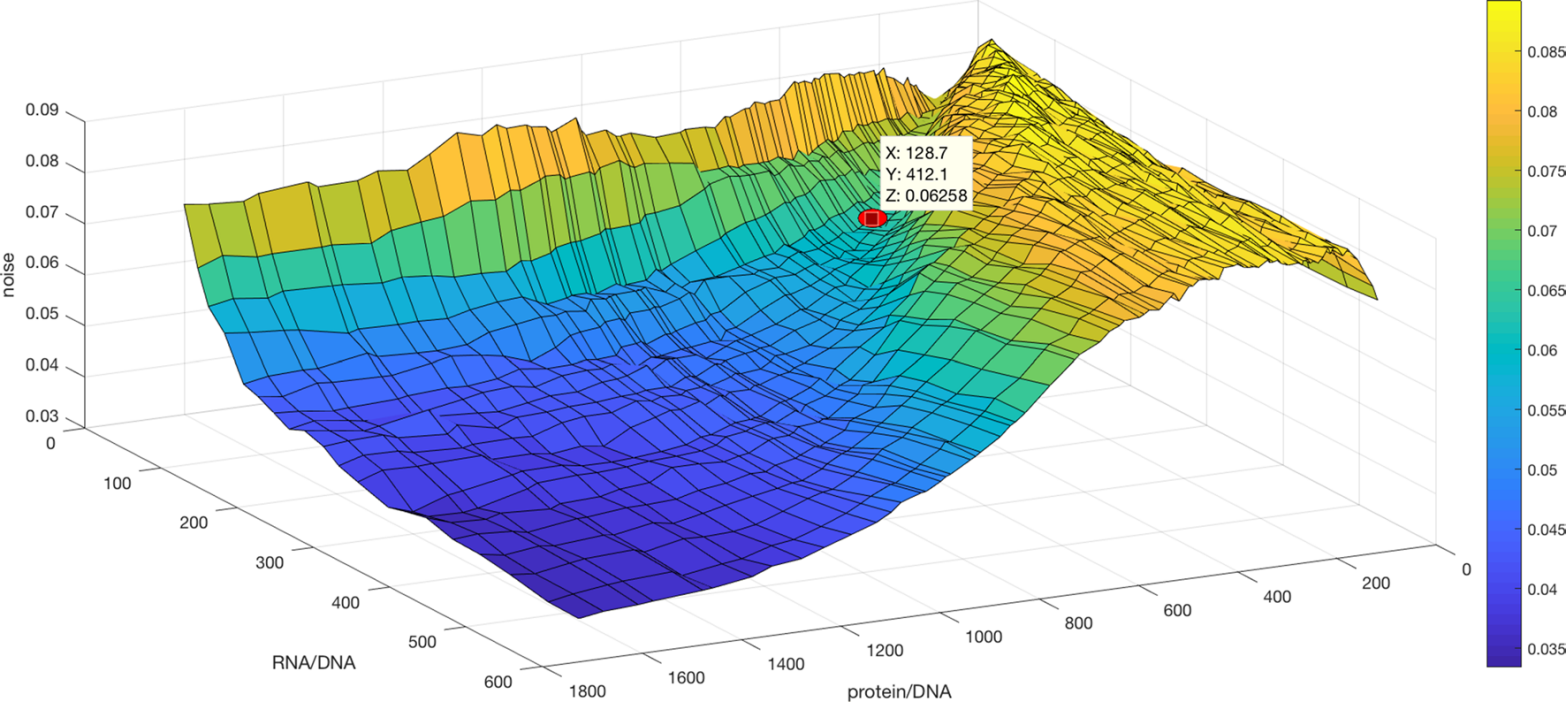
Stochastic noise in CCG-2 usually decreases with increases in hypothesized ratios of RNA/DNA and Protein/DNA within a single cell. The total stochastic noise ${\sigma }_{f}^{2}$ averaged over frequencies (f) in CCG-2 expression is computed from 1,024 Gillespie trajectories from the best model in S Table 1 with a ${\chi }^{2}$ = 2,671.95. The best model selected was one with minimum chi-squared statistic based on the Likelihood in Eq. (2) for the D/D and 3 L/D experiments from an accumulation run based on 12 genetic algorithms in Table 1. The red dot denotes the experimentally determined ratios previously^14^ and corresponds to RNA/DNA and protein/DNA ratios of 128.7 and 412, respectively. The model with the best chi-squared statistic in the accumulation run was modified to different RNA/DNA and Protein/DNA ratios for each point on the grid above. A total of 1,024 Gillespie trajectories were generated for each model on the grid. The variance in the 1,024 resulting periodogram height was computed for each sample frequency ${f}_{l}$ . These variances were summed over all frequencies to produce the noise on the z-axis. The plot was created in MATLAB_R2018B (https://www.mathworks.com/products/matlab.html).

In previous work evidence was presented that the RNA/DNA and protein/DNA ratios for *ccg-2P:mCherry* strain set the levels of stochastic intracellular noise in a cell, and hence these ratios were measured^14^. They continue to serve a similar role in a system with light entrainment (Fig. 5). As the RNA/DNA and protein/DNA ratios are increased, leading to larger amplification in RNA counts and protein counts, there was a general decrease in the noise in the system (Fig. 5). Imagine the red dot as a ball; from most places on the surface the ball rolls to the lowest point in the front left corner of Fig. 5. The only caveat is a shallow ridge at low protein/DNA ratios. The relationship between the stochastic intracellular noise and the RNA/DNA and protein/DNA ratios is not in and of itself surprising^16,20^; however, exploiting this relationship to determine “size of the cell” appears to be new^14^. This ability to determine empirically the “size of the cell” is why the relation in Fig. 5 is presented. In this way these ratios can be used to manipulate the level of stochastic intracellular variation. These ratios were experimentally determined (red dot) previously to set the level of noise in each cell^14^.

### The phase variation over time between cells provides an independent test of the goodness of fit to that predicted by the best model

There are three ways to characterize periodic processes, by their period, amplitude, and phase^6^. The period and amplitude are captured in the periodogram or power spectrum (e.g., Fig. 4), which was used to fit the model ensemble in Eq. (2). The remaining measure, phase, is functionally independent of the periodogram and was not used to fit the stochastic network to the single cell data and hence is available to test goodness of fit of the stochastic network^14^.

There are a variety of ways to measure phase, as described in an accessible introduction to phase measures^31^. In addition to its independence from the periodogram, the phase measure used here and elsewhere was used to assess whether or not synchronization is taking place between single cells experiencing a common driving light signal^5,30,42–45^ (see “Materials and methods” for a definition of phase).

**Figure 6:**
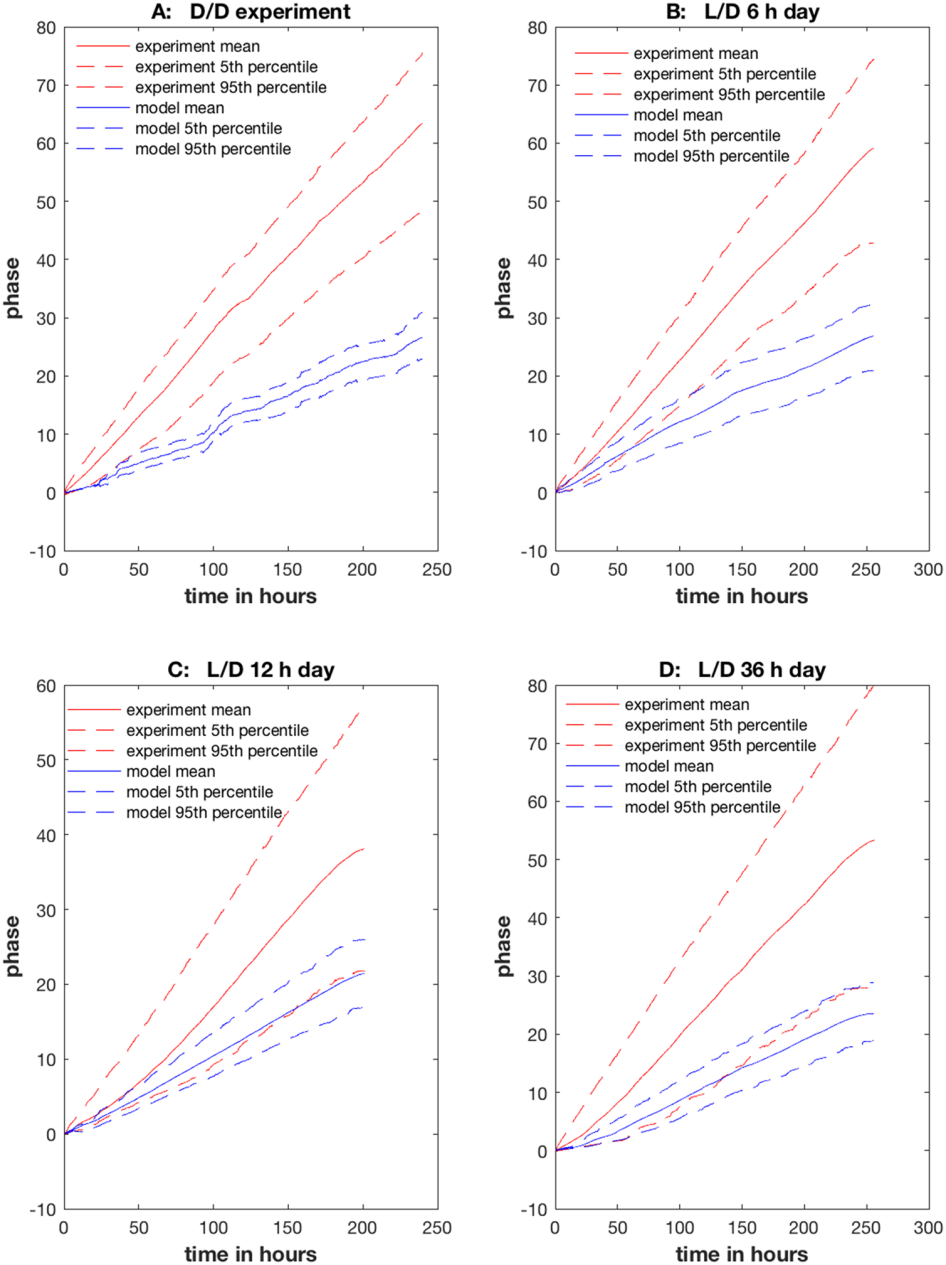
The phase plots as a function of time indicated that there are limitations on goodness of fit for the D/D experiment and 6 h day L/D experiment. The 95th percentile, the mean, and the 5th percentile of the phase for all cells are graphed for each experiment (red) and the model (blue) computed from 1,024 Gillespie Trajectories from the best model in Supplementary Table S1. Single cell trajectories for data and model are summarized under the: (**A**) D/D experiment; (**B**) 6 h day L/D experiment; (**C**)12 h day L/D experiment; (**D**) 36 h day L/D experiment. The plots were created in MATLAB_R2018B (https://www.mathworks.com/products/matlab.html).

To provide an independent test of the stochastic network in Fig. 1, the average phase with percentiles was computed over time for cells in all four experiments both for the data and for the model (using 1,024 generated single cell Gillespie trajectories) (Fig. 6). For the D/D and 6 h day L/D experiments the goodness of fit failed at the 75 h and 125 h mark, as the data (in red) drifted beyond the percentile bands of the model (blue). In contrast, the percentiles of phase for model and data remained overlapping for the 12 h day and 36 h day L/D experiments.

The phase plots also provided information about the cellular clocks in single cells. Phase plots for both the model and data in the 12 h day and 36 h day L/D experiments were bent and hence demonstrated synchronization to the driving light signal. Also, all plots showed increased variation in phase over time, capturing the tug of war between stochastic intracellular noise generating phase variation and light producing changes in phase synchronization and hence the phase mean. The degree of linearity of the D/D and 6 h day L/D experiment (r = 0.9995 and r = 0.9998, P < 0.0001) would also suggest that a sinusoidal approximation would be a good one^31^. The fact that the D/D and 6 h day L/D experiments did not demonstrate a nonlinear response in time and hence synchronization was consistent with the synchronization measures for the D/D (Kuramoto K = 0.08 ± 0.0026) and 6 h day L/D (K. = 0.30 ± 0.0066) experiments being smaller than those for the 12 h day L/D (K = 0.42 ± 0.0076) and 36 h day L/D (K = 0.33 ± 0.0069) experiments^5^. For example, the maximum in light synchronization was measured to take place with a 12 h day, which also show a nonlinear response in the phase curve over time^5^. This phase synchronization becomes even more pronounced when the number of cells is scaled up to over 40,000 cells and when the cells are allowed to communicate^5^.

**Figure 7:**
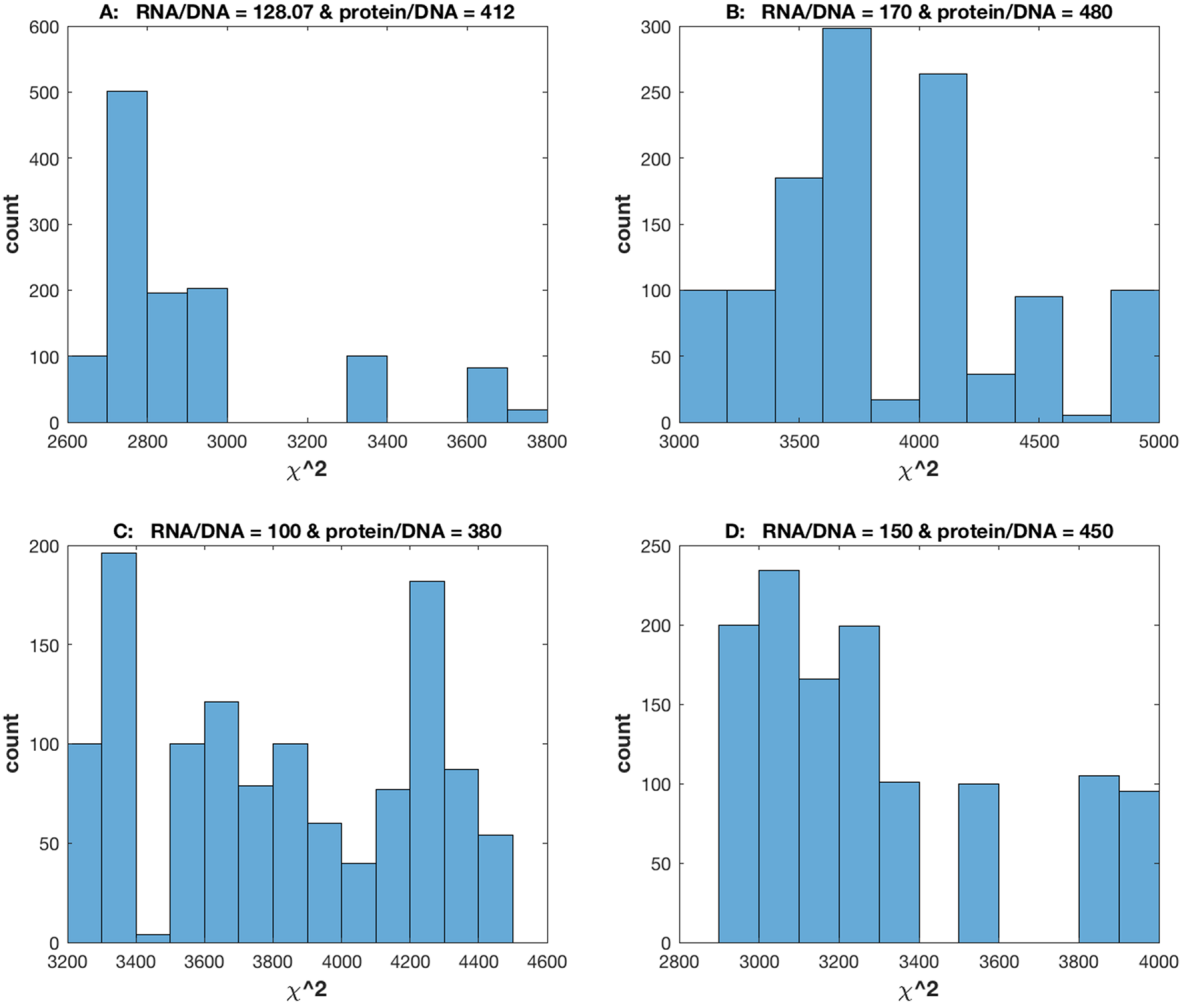
The goodness of fit as measured by the chi-squared statistic in (2) is robust to variation in the ratios of RNA/DNA and protein/DNA and hence the stochastic intracellular noise from Fig. 5. Histograms of the chi-squared statistics of 1,200 models in the accumulation run for determining the chi-squared empirical distribution are shown. The ratios of RNA/DNA and protein/DNA used in each of the 1,200 models was, respectively: (**A**) 128.07 and 412; (**B**) 170 and 480; (**C**) 100 and 380; (**D**) 150 and 450. A description of how the ratios are varied without altering the rate constants is shown in the “Materials and methods”. The plots were created in MATLAB_R2018B (https://www.mathworks.com/products/matlab.html).

Stochastic networks have one other dimension to goodness of fit absent in deterministic network models. Having determined what the “size of a cell” is by measuring the RNA/DNA and protein/DNA ratios in Fig. 5, it is natural to ask how these ratios affected the goodness of fit of the model periodograms to the average of the observed single cell periodograms. These ratios were varied substantially about their measured values to see the effect on goodness of fit (Fig. 7). The fitting of the D/D data would leave us to hypothesize that the goodness of fit would be robust to variation in the level of stochastic intracellular noise captured by these ratios^14^.

We found the distribution across the fitted model ensemble was quite robust to variation in these ratios (Fig. 7). This robustness property can be predicted from the Chemical Langevin Equations that approximate the stochastic network in Fig. 1^14^.

The robustness of the goodness fit plots as assessed by the phase plots (Fig. 6) was also examined for several values of the RNA/DNA and protein/DNA ratios (Fig. 6, Supplementary Figs. S4–S5) that increased the stochastic intracellular noise without substantial alteration in fit to the periodogram. By increasing the noise with ratios of 60 and 300 or 100 and 380 for the RNA/DNA and protein/DNA ratios without changing the rate constants (see “Materials and methods”), the phase plots of the models could be aligned better with the experimental phase plots (Supplementary Figs. S4, S5) than with using the experimental ratios of 128.07 and 412. These new ratios left the goodness of fit to the periodogram intact (Fig. 6C), but the phase plots were a little noisier (Supplementary Figs. S4–S5).

### Is there one intermediate optimum in the oscillatory signal as a function of the stochastic intracellular noise?

The heart of the experiments and calculations in this paper is to examine whether or not the working model in Fig. 1 displays Stochastic Resonance, i.e., a nonlinear relation between the signal/noise ratio captured in the power spectra and the stochastic intracellular noise in the system^9^. The noise is varied by altering the RNA/DNA and protein/DNA ratios in the cell about the measured values. High values of the ratios imply low noise while low ratios imply high noise in Fig. 5.

**Figure 8:**
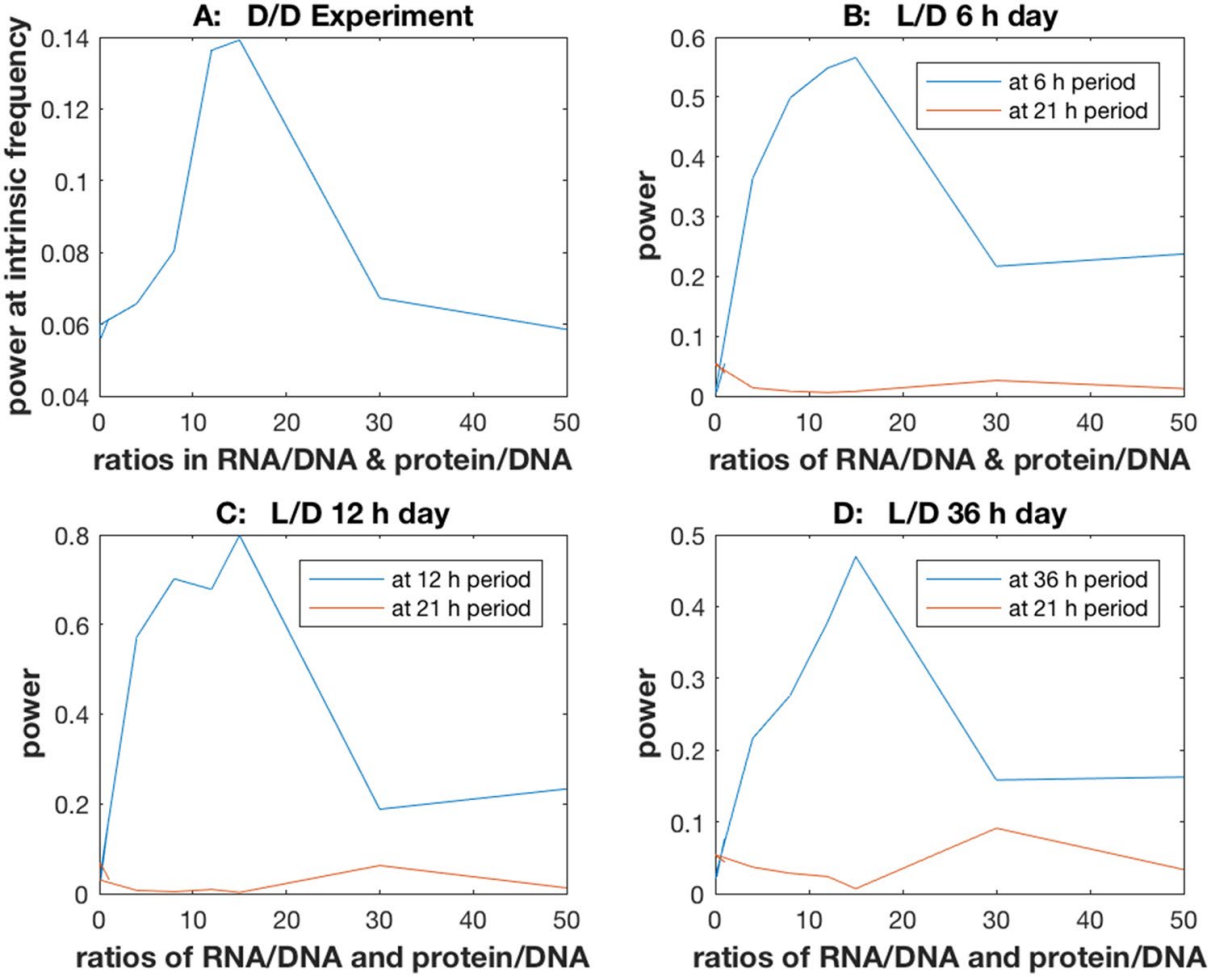
The power at the driving frequency or intrinsic frequency for a cellular oscillator is a nonlinear function of the stochastic intracellular noise and take a maximum at a ratio of 15 for RNA/DNA and protein/DNA for all four independently conducted experiments. The stochastic intracellular noise was varied by multiplying the RNA/DNA and protein/DNA ratios observed in such a way as not to change the rate constants by a ratio of: 1/7, 1/10, 1/12, 1/40, 1/100, 1/170, 4, 8, 12, 15, 30, or 50. (**A**) D/D experiment; (**B**) L/D 6 h day; (**C**) L/D 12 h day; (**D**) L/D 36 h day. The model used to generate the power values above is the best fitting model in Supplementary Table S1. The power values are derived from the periodograms in Supplementary Fig. S6. For the L/D experiments the power at the intrinsic frequency of a cellular oscillator is added as a control. The plots were created in MATLAB_R2018B (https://www.mathworks.com/products/matlab.html).

The results of this experiment are shown in Supplementary Fig. S6. Reducing the ratios by a constant factor generally decreases the power at the intrinsic frequency (Fig. 8a) or at the driving frequency (Fig. 8b–d). In contrast as the ratios are increased, there is a spike in the signal at the resonance, which then fades away as the ratios are increased further. These changes in the ratios were done to preserve the rates constants at the best fitting model in Supplementary Table S2 while varying the stochastic intracellular noise^14^. For the 36 h day the ratios had to be increased further to see the signal to noise ratio diminish in Supplementary Fig. S6.

The results are more easily summarized in Fig. 8. The ratios in Fig. 5 are varied from low (high noise) to high (low noise), and the power in each experiment is presented at the intrinsic frequency of the cellular oscillators (~ 21 h) or at the driving frequency (~ 6 h, ~ 12 h, or ~ 36 h, depending on the L/D experiment). There is a clear nonlinear relation for each day that peaks at the same ratio of 15 X the original ratios (128.07 for RNA/DNA and 412 for protein/DNA). The intrinsic frequency is plotted as a control (in red) for the L/D experiments.

**Figure 9:**
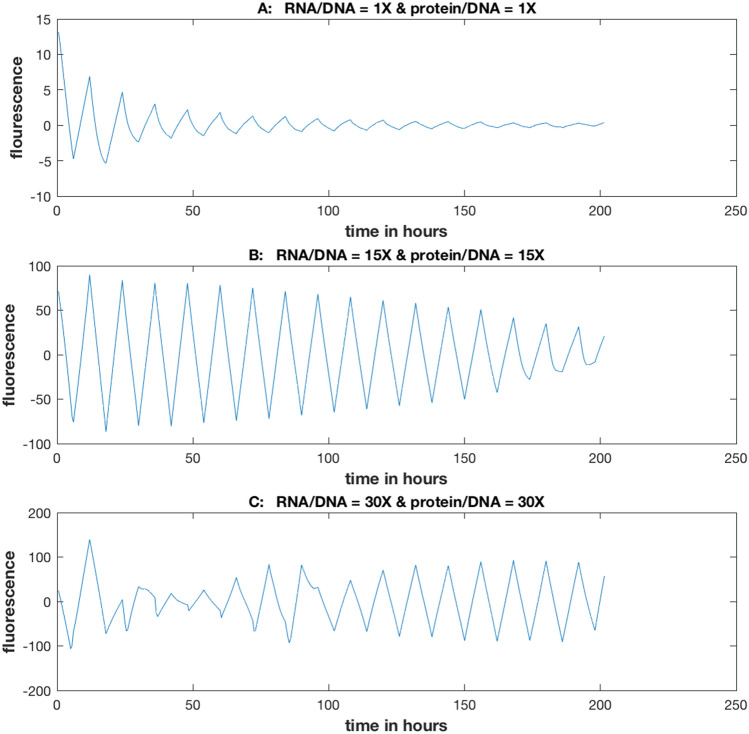
The effects of stochastic intracellular variation at the resonance was to amplify the circadian signal, but away from the resonance the signal was degraded. These FRQ trajectories are averages over 1,024 Gillespie trajectories at the best model (Supplementary Table S1). The y-axis is the predicted number of the FRQ oscillator protein over time. The RNA/DNA and protein/DNA ratios are at ×1, ×15, and ×30 of their measured values of 128.7 and 412, respectively. The stochastic intracellular noise was varied by changing the initial molecular counts as in Fig. 8. The plots were created in MATLAB_R2018B (https://www.mathworks.com/products/matlab.html).

The effects of stochastic intracellular noise on the average Gillespie trajectory are shown for a 12 h L/D cycle (Fig. 9). As can be seen, away from the Stochastic Resonance there is a degradation in the circadian signal, and at the stochastic resonance there is an amplification of the circadian signal. In fact the light and the stochastic intracellular noise lead to highly synchronized behavior at the resonance to reinforce the oscillations (see video). As a consequence at the resonance a high Kuramoto K order parameter is achieved. This is a classic example of stochastic resonance in a biological system^9^. These striking differences in the circadian oscillations arise between cells that are genetically identical!

## Discussion

There are three hypotheses about how oscillations arise at the single cell level. One hypothesis is that stochastic noise contributes to the oscillations, a theory known as Stochastic Resonance^9^. Two teams indicated how Stochastic Resonance could serve as a mechanism to generate such oscillations^46,47^ and possibly to synchronize cellular oscillators^48^. A second hypothesis is that there is a chemical signal through quorum sensing by cellular clocks that is involved in synchronization of single cell oscillators^5,7^, thus explaining circadian rhythms on the macroscopic scale of 10^7^ cells. A third possibility is cell cycle gating of the single cell oscillators to reinforce the oscillations^10,11^. Under this third possibility there may be no specific genes that induce coordination between different single cell oscillators as for example, in a quorum sensing hypothesis of cell-to-cell communication. The advantages of this study using the model system, *N.* *crassa*, is that the single cell environment can be set up to test each of these hypotheses individually using microfluidics^12^**.** Here a flow focusing, droplet generating microfluidics device was used to isolate *N.* *crassa* cellular oscillators for testing Stochastic Resonance^5^. The microfluidics device isolated cells in droplets to prevent any form of chemical communication, as under a quorum sensing hypothesis^5^. The media were selected as well so that there was no cell division to eliminate cell cycle gating as a hypothesis^6^. The model system was exploited in such a way as to be able to take advantage of light entrainment of isolated *N.* *crassa* single cell oscillators^5^, an advantage not present in other model clock systems, such as mammalian^22^ or Cyanobacterial^21^ model clock systems. While we have provided evidence that Stochastic Resonance may be operating by itself, that does not rule out the possibility of cell cycle gating or quorum sensing operating in conjunction with Stochastic Resonance. Under other experimental settings, such as when cells can communicate within droplets^6^, it will be interesting to study the joint effects of Stochastic Resonance with these other mechanisms.

It was possible to demonstrate here strong support for a neutral model without any cellular communication using both light entrainment experiments and D/D experiments to specify a model ensemble in Fig. 1 describing cellular clocks (Fig. 4). In four independent light entrainment experiments the model ensemble was able to capture the period and amplitude behavior of the single cell oscillators from a 6 h L/D cycle to a 36 h L/D cycle at the single cell level (Fig. 4). The highly successful fitting was robust to variation in “cell size” present in stochastic networks, as captured in the proxies for cell size, the measured RNA/DNA and protein/DNA ratios (Fig. 8). The fitted model ensemble displayed the same stochastic resonance across all four D/D and L/D experiments as the stochastic intracellular noise was varied through the RNA/DNA and protein/DNA ratios (Fig. 8). This neutral model with Stochastic Resonance then is a promising framework for testing whether or not Stochastic Resonance can explain by itself the origin of circadian rhythms on a macroscopic scale from the cellular clocks operating on a microscopic scale.

There were some limitations to the neutral model supporting Stochastic Resonance. The periodograms (Fig. 4) used to fit the stochastic network in Fig. 1 captured the amplitude and period variation in cellular clocks remarkably well (${\chi }^{2}$ = 2,671.95 across 953 frequencies from four periodograms and with 35 model parameters with a chi-squared statistic per data point of $\frac{{\chi }^{2}}{n}=2.80$), but the periodograms are functionally independent of the phase variation^6,31^, when measured by Hilbert Phase^30^. The phase measure used here and derived from the Hilbert Phase^30^, by virtue of its functional independence of the periodogram^31^, was used to test goodness of fit to the single cell experiments (Fig. 6). The results were that for the L/D 12 h and 36 h day the phase of the model ensemble and single cell data over time were consistent with each other (Fig. 6C,D); however, there were some departures from model ensemble predictions of phase over time after 75 h (Fig. 6A) for a D/D experiment or 125 h for the single cell data (Fig. 6B) for 6 h L/D experiment. As can be seen in the fan shape of the percentile bands for phase over time (Fig. 6), there is a tug of war between the substantial role of stochastic intracellular noise generating phase variation between cells and the effect of the light signal on the mean phase of the cellular clocks. One possible explanation for the departure may be that stochastic intracellular variation is winning the war as the system evolves in time, causing the phase of single cells to drift outside the percentile bands of the model ensemble when the synchronization with the light signal is weaker^5^ in Fig. 6A,B. The fit could be improved by using RNA/DNA and protein/DNA ratios that were smaller than measured (Supplementary Figs. S4–S5) with the result that the additional stochastic intracellular noise aligned the phase plots of the models better (Supplementary Fig. S4–S5) with the observed phase plots.

The fact that noise plays such a significant role in generating phase variation raised the possibility that the behavior of cellular clocks may be fundamentally different from the rules of clocks at the macroscopic scale of 10^7^ cells/ml^18^. We tested this possibility by examining the fitted rate constants derived from single cell data. The result was excellent agreement with the characterized dynamics on the macroscopic scale^18,19^ in Table 2. For example, the prediction that the lifetime of the *wc-1* mRNA being long as measured^18^ on a macroscopic scale, held up on a microscopic scale when light entrainment data for single cells were added (Table 2). At this stage there was little evidence for cellular clocks playing by different rules than those at the macroscopic scale of 10^7^ cells.

There are a variety of kinds of resonances that could be at play in the circadian system of single cells of *N.* *crassa*^5^. For example, the resonance could be due to noise acting near a single excitation state in the model^49^, as in the phase resetting of cyanobacterial cells^24^ or alternatively, due to noise moving the system from one equilibrium point to another as in a bistable switch^49^. One characteristic of a stochastic resonance, whether it be introduced as in a signal processing tool or naturally occurring, is the presence of at least one stochastic switch^50^. Sriram and Gopinothan^51^ were among the first to hypothesize such a stochastic resonance in the *N.* *crassa* circadian system. The basis for such a stochastic switch in Fig. 1 lies in the stochastic switching on or off of the oscillator gene *frq* or the *ccg* gene^5^. The one or few copies of genes themselves in Fig. 1 provide the basis of the stochastic switching mechanism. Further experimental and theoretical studies of the model (Fig. 1) are required to characterize the resonance. For example, *N.* *crassa* single cell behavior through microfluidic experiments to examine transcriptional bursting^52,53^ and calculations of the mean amplitude, period, and phase of the model^54^ will be needed to arrive at details of the resonance mechanism. Some of this work has already begun on single cell measurements of the mammalian SCN in a phenomenological way by fitting simplified damped or self-sustained oscillators to single cell data on circadian rhythms of the SCN^55^.

Another major limitation of the work here is the focus on single conidial cells; however, the predominant life stage of *N.* *crassa* is the filament or hyphae in which cell nuclei share a common synctitium that is constantly expanding as the hyphae grow^56^. Much of the work on the clock in *N.* *crassa* focuses on this life stage in “race tubes”^57,58^. This shared cytoplasm between nuclei in hyphae raises the interesting possibility of other new forms of communication between nuclei as they move down a race tube, such as through transvection and intra-filament diffusion of signaling molecules^56^. New kinds of microfluidic devices will be required to study how these communication mechanisms synchronize nuclei in hyphae^59,60^.

There are other features of the genetic network (Fig. 1) that are hypothesized to mediate the effects of stochastic intracellular noise other than through a resonance^16^. Andreas Wagner^61^ has demonstrated simple two-gene circadian oscillators with interlocking regulatory connections are more likely to be robust in period by an MCMC analysis. Liu et al. provided experimental evidence on a macroscopic scale that the positive feedback loop by FRQ on *wc-1* mRNA involving (C1)^62^ in Fig. 1 functions to provide stability and robustness to the clock. It would be interesting to know what effect stochastic intracellular noise has on single cell circadian oscillations when the positive feedback loop in Fig. 1 is removed.

Yu et al.^18^ reviewed experimental evidence for each reaction in the topology in Fig. 1 on a macroscopic scale. As more data are gathered, it may be necessary to alter the topology of the network in Fig. 1 as another parameter in the model. Al-Omari et al.^63^ developed ensemble methods to identify the topology of the network using the supernet. It may be possible to extend these supernet methods to the single cell level using single cell sequencing^64^, allowing a reassessment of the topology in Fig. 1 at the single cell level.”

What made the results here possible was the development of new fitting methods for stochastic networks^14^ in particular and for ensemble methods in general^25^. A longstanding problem (20 years) for ensemble methods applied to oscillatory systems has been the inability to generate successfully a fitted model ensemble without an initially informed guess as to the rate constants and initial species concentrations^25^. We were so limited in the development of finding an ensemble of stochastic networks in Fig. 1 using existing ensemble methods with parallel tempering in Fig. 3^14^. Here by the introduction of genetic algorithms into the equilibration phase of a Markov Chain Monte Carlo reconstruction of the likelihood for a stochastic network in Equation (2), a random initialization of genetic algorithms (Table 1) outperformed existing parallel tempering methods starting with an informed guess as to model parameters^14^. These genetic algorithms also yielded solutions in less time by an order of magnitude (Fig. 3). As a result the speedup of the genetic algorithms could be used to generate evidence that a global optimum in the fit of the model ensemble was achieved (Fig. 3B).

The ability to fit stochastic networks to single cell data quickly and efficiently suggests new microfluidics experiments to test the physical hypothesis of Stochastic Resonance in biological systems. The prediction of only one stochastic resonance across light entrainment experiments in single cells provides a unique opportunity to test the Theory of Stochastic Resonance in a biological system^9^.

## Conclusions

We have developed an ensemble method for stochastic networks using a particular class of genetic algorithms called Particle Swarm Optimization methods that is successful in fitting a stochastic clock network ensemble to single cell data on *Neurospora* *crassa* under a variety of Light/Dark conditions along with the Dark (D/D) condition. The model fitted only displays one stochastic resonance (at one ratio of the RNA/DNA and protein/DNA) for a variety of light regimes. That is, as the stochastic intracellular noise is varied there is a unique optimum in the signal to noise ratio in the circadian rhythm. When there is a departure from this resonance, there is a dramatic degradation in the circadian rhythm even though all cells are genetically identical. The right level of stochastic intracellular noise is essential for strong circadian rhythms.

## Materials and methods

### Single cell data of *N.crassa*

The single cell data from four experiments are used to evaluate the stochastic clock network in Fig. 1. The cells in these experiments are equipped with an mCherry recorder under the control of a *clock-controlled* *gene-2* promoter (*ccg-2P*)^33^*.* This fluorescent mCherry strain is referred to as MFNC9^33^. Each of the four experiments involved isolating over 1,000 cells in individual droplets, synchronizing cells initially with 26 h of light, and then observing their fluorescence every half hour for at least 10 days^6^. Four experiments were conducted, one in the dark (D/D) and three under 6 h, 12 h, or 36 h L/D regime with equal amounts of light and dark^6^. The D/D data are available^14^, and the L/D entrainment data are available at the IEEE Dataport, https://ieee-dataport.org/documents/single-cells-neurospora-crassa-show-circadian-oscillations-light-entrainment-temperature.

### Rescaling from deterministic model units to stochastic molecular number units

A method for rescaling initial concentrations and reaction rates of a deterministic network to molecular counts and reaction rates of a stochastic network was described previously^17^.

The network is divided into small subnetworks called “boxes” such that there is usually no net flow of molecules between different boxes in Fig. 1B. Then the concentration of each species in a box is scaled by a certain factor. The reaction rates are then scaled so that the network dynamics are not changed.

### Rescaling with the RNA/DNA and protein/DNA ratios without changing the network dynamics

The model above specifies the Master Equation, which describes how the counts of molecular species in Fig. 1B change over time^15^. The Master Equation can be approximated by the Chemical Langevin equation^20^, which consists of two components, a deterministic term and a noise term. The deterministic term corresponds to a system of ordinary differential equations. The first term is required to be invariant under rescaling by the RNA/DNA and protein/DNA ratios to leave the network dynamics invariant. Consider one component of the deterministic term, namely the L3 and D6 reactions in dotted box d of Fig. 1B:

$${frq}^{r1} \underset{L3}{\Rightarrow }FRQ+{frq}^{r1}, FRQ \underset{D6}{\Rightarrow } \oslash$$

The contribution to the dynamics of FRQ by this reaction is:

$$\frac{d[FRQ]}{dt}=L3\left[{frq}^{r1}\right]-D6\left[FRQ\right]$$

The ratios of RNA/DNA (${R}_{RNA:DNA}$) and protein/DNA (${R}_{Prot:DNA}$) are measured experimentally or changed to vary the stochastic intracellular noise. If the ratios are changed, then the scales of RNA and protein counts change as well so that:

$$\frac{d{R}_{Prot:DNA}\left[FRQ\right]}{dt}={L3}_{new}{R}_{RNA:DNA}\left[{frq}^{r1}\right]- {D6}_{new}{{R}_{Prot:DNA}}_{2}\left[FRQ\right]$$

This becomes:

$$\frac{d\left[FRQ\right]}{dt}={L3}_{new}\frac{{R}_{RNA:DNA}}{{R}_{Prot:DNA}}\left[{frq}^{r1}\right]- {D6}_{new}\frac{{R}_{Prot:DNA}}{{R}_{Prot:DNA}}\left[FRQ\right]$$

One way that the dynamics remain unchanged is if:

$$L3={L3}_{new}\frac{{R}_{RNA:DNA}}{{R}_{Prot:DNA}} \; \mathrm{ or } \; {L3}_{new}=L3\frac{{R}_{Prot:DNA}}{{R}_{RNA:DNA}} \; and \; {D6}_{new}= D6.$$

In this way by stepping through all of the dotted boxes in Fig. 1, all 23 reaction rates can be rescaled to preserve the original dynamics when the RNA/DNA and protein/DNA ratios are changed to vary the noise in the stochastic network. The rescaling for some other dotted boxes is illustrated in an earlier work^14^.

### Stochastic simulation algorithm-direct method

Gillespie developed several methods for simulating exactly and approximately the trajectory of a stochastic network^6,15,65^. Here we describe his exact direct method implemented previously and currently on Graphical Processing Units or GPUs^14^. The use of GPUs has been critical to implementing the ensemble methods at the single cell level and macroscopic level^63,66^. Under the direct method Gillespie demonstrated that knowing the current state of the network at time t, the distribution of times of the next reaction at time $t+\tau$ and the probability of each reaction can be computed by simulation. The result of the direct method is to know the distribution of states of the network at time $t+\tau$. If this procedure is applied sequentially in time, then a whole stochastic trajectory of the system is constructed. Each of the trajectories is called a Gillespie Trajectory and represents the history of a single cell.

Here is how the direct method provides the computation of the state probabilities from the N (= 12) initial conditions ${X}_{i}$ for the ith molecular species in the network and the M (= 23) rate constants ${k}_{i}$ in the network. All of the parameters in the stochastic model are listed in a $\theta$-vector defined by $\Theta =\left\{{X}_{1}\left(0\right),{X}_{2}\left(0\right),\dots ,{X}_{N}\left(0\right),{k}_{1},\dots ,{k}_{M}\right\}$. Thus, we obtain an exact distribution of the state of the network at time $t+\tau$. Given the model parameters $\Theta$ and a final time $T$, the following iterative loop is performed to generate a Gillespie trajectory for a single cell:

1. The system is initialized by setting $t=0$ and $X=x=\left\{{x}_{1}\left(0\right),{x}_{2}\left(0\right),\dots ,{x}_{N}(0)\right\}$.
2. The propensities of a reaction, ${a}_{j}\left(x\right), j=1,...M$, and their sum ${a}_{0}=\sum_{j=1}^{M}{a}_{j}(x)$ are calculated. The propensities are: given the current state x of the system at time, the propensity is the probability of reaction j in an infinitesimal time interval t + dt^15^.
3. A random time step value to the next reaction, $\tau$, is drawn from an exponential random variable with mean $1/{a}_{0}(x)$. The type of the next reaction, ${j}_{next}$, is randomly drawn with probabilities $\frac{{a}_{j}\left(x\right)}{{a}_{0}\left(x\right)}, \quad j=1,\dots M.$.
4. The state $X$ is updated assuming reaction ${R}_{{j}_{next}}$ took place. the time, $t=t+\tau$, is updated as well.
5. If $t<T$ return to step 2; else, stop.

For example, the propensity of the reaction to form WCC at time t is proportional to:

$$C2{x}_{WC-1}(t){x}_{WC-2}(t)+C2{f}_{IL}{x}_{WC-1}(t){x}_{WC-2}(t)s(t)$$

In that the number of WC-2 molecules (${x}_{WC-2}$) is kept constant, it was absorbed into C2. The factor s(t) is changing exogenously to the Master Equation. Only the first term is present in the dark, and the second term is added in each light window.

The Direct method yields a Gillespie trajectory of network states $\left\{x\left({t}_{0}\right),x\left({t}_{1}\right),\dots x({t}_{k})\right\}$ in the time interval $[0,T]$. The Gillespie trajectory describes the state of a cell over the time interval $[0,T]$. It is understood that the temporal sequence satisfies $0={t}_{0}<{t}_{1}<\dots <{t}_{k}<T$ with ${t}_{i}$,i = 1,…,k, being the reaction times of the reactions that fire before $T$. Such a trajectory completely identifies the network state of a single cell at any time in the interval $[0,T]$.

### A bias-corrected periodogram averaged over cells is the model-fitting criterion

Detector noise has been argued to be an important consideration in network reconstruction^67^. In previous work it was found that separating the detector noise in measuring fluorescence of single cells of MFNC9 from the stochastic intracellular noise was essential for fitting the clock stochastic network to the D/D single cell data because the detector noise introduced substantial bias into the periodogram^14^. It was possible to separate the detector noise from the stochastic intracellular noise by replacing living cells by doped beads in the same microfluidics experiments^6^. A model was developed to measure the detector noise from the doped bead experiments. The assumptions of the model were that: (1) the total noise in fluorescence of an individual MFNC9 cell could be decomposed additively into detector noise and stochastic intracellular noise; (2) the two noise components were statistically independent; and (3) the noise in the denominator of the Rhodamine-B normalized time series could be neglected (see denominator in Equation S11 of the supplement^6^). Under these assumptions it was possible to calculate the bias in the periodogram by a propagation of errors calculation from the detector noise^6^.

The fluorescent observations on single cells of the mCherry strain MFNC9^33^ were made on an equally spaced time grid of L observations every half hour for all four experiments from time 0 to time T, the duration of the experiment. What varied from experiment to experiment was the number of time points L on K cells. For example, for the L/D entrainment experiment with a 6 h day, L = 512. The observation time of the jth observation along the grid for each of these four experiments is ${t}_{j}=\left(j-1\right)\frac{T}{L}$. These L time points of fluorescence were Rhodamine B normalized to remove uncontrolled biases, detrended with a moving average^35^ to filter out high frequency noise, and then transformed into the frequency domain using a Fast Fourier Transform^68^ to evaluate the significant frequencies (such as one that might correspond to a circadian rhythm) in the fluorescent trajectory. The uncorrected periodogram $Q\left({f}_{l}\right)$ of each cell derived from the Fast Fourier Transform is the power as a function of the sample frequencies ${f}_{l}=\frac{l}{T}$, *l* = 0,…, [*L*/2]. The detector noise contribution to the periodogram at frequency ${f}_{l}$ was then shown previously to be^6^:

1 $${\left({\sigma }_{l}^{e}\right)}^{2}=\frac{2{\sigma }_{\epsilon }^{2}}{KL}\left[\langle Q\left({f}_{l}\right)\rangle {\gamma }_{Q}\left(l\right)+Re\left(\langle R\left({f}_{l}\right)\rangle {{\beta }_{Q}\left(l\right)}^{*}\right)\right]-\frac{{\sigma }_{\epsilon }^{4}}{K{L}^{2}}\left[{\left|{\gamma }_{Q}\left(l\right)\right|}^{2}+{\left|{\beta }_{Q}\left(l\right)\right|}^{2}\right],$$

Brackets are used to denote expectations over the whole population of single cells. For example, $\langle Q\left({f}_{l}\right)\rangle$ and $\langle R\left({f}_{l}\right)\rangle$ are the population means of the average periodogram and average squared Fourier transform of the observed Rhodamine B-normalized, detrended fluorescence time series, respectively. By varying the light intensity in a series of microfluidic experiments the variance in fluorescence of the beads was then used to estimate the variance of the fluorescence signal ${\sigma }_{\varepsilon }^{2}$ due to the detector noise averaged over all cells and time points. This variance was determined experimentally by varying the incident light intensity and measuring the resultant variance in fluorescence of fluorescent beads replacing cells in a microfluidics experiment identical to that used for cells^6^. The functions ${\gamma }_{Q}\left(l\right)$ and ${\beta }_{Q}\left(l\right)$ are determined by weights used in the moving-average detrending process^35^, a standard for the literature. The functions ${\gamma }_{Q}\left(l\right)$ and ${\beta }_{Q}\left(l\right)$ do not depend of the observed fluorescence signals.

The detection noise created bias in the observed periodogram in (1) and was removed from the raw periodogram by subtracting.

$${Q}^{bias}\left({f}_{l}\right)=\frac{{\sigma }_{\epsilon }^{2}}{L}{\gamma }_{Q}\left(l\right).$$

The model generated periodogram (computed by simulating a 1,024 Gillespie trajectories and their associated average periodogram) was fitted to the bias corrected periodogram $\langle Q\left({f}_{l}\right)-{Q}^{bias}\left({f}_{l}\right)\rangle$^39^. The quantity $\langle Q\left({f}_{l}\right)\rangle$ is the average periodogram over *K* cells calculated at frequency ${f}_{l}$ and $\langle {Q}^{model}\left({f}_{l}\right)\rangle ,$ the average periodogram of 1,024 Gillespie trajectories calculated at ${f}_{l}$ .

The criterion used to select the ensemble fitting the single cell trajectories was then

2 $${\mathbb{Q}}_{bias-free}(\Theta )={\Omega }^{-1}\prod_{l}\frac{1}{\sqrt{2\pi {\left({\sigma }_{{f}_{l}}^{c}\right)}^{2}}}exp\left(-\frac{{\left(\langle Q\left({f}_{l}\right)-{Q}^{bias}\left({f}_{l}\right)-{Q}^{model}({f}_{l}\rangle \right)}^{2}}{2{\left({\sigma }_{{f}_{l}}^{c}\right)}^{2}T}\right)=exp\left(-{\chi }^{2}/2\right){\Omega }^{-1}\prod_{l}\frac{1}{\sqrt{2\pi {\left({\sigma }_{{f}_{l}}^{c}\right)}^{2}}}$$

with ${\left({\sigma }_{{f}_{l}}^{c}\right)}^{2}= {\sigma }_{{f}_{l}}^{2}-{\left({\sigma }_{{f}_{l}}^{e}\right)}^{2}$. The quantity T is temperature and is set during a Markov Chain Monte Carlo Procedure called parallel tempering described below to recover the ensemble from the single cell data; in other Monte Carlo methods it may be set to 1. The ensemble in (2) in this case is the bias-free distribution of the periodogram averaged over more than 1,000 single cells. The justification of the normal distribution assumption about the average periodogram computed over > 1,000 single cells in (2) is the Central Limit Theorem^69^. See^6^ for details. Since the experiments were done independently, the ensemble for all four experiments simultaneously can be obtained by multiplying the expressions (2) for each experiment to obtain the joint ensemble for all 4 experiments.

## Calculation of phase

MATLAB scripts are available in GitHub to examine the phase measures below^31^. Consider a fluorescent series of measurements on MFNC9^33^ denoted by $x(t)$. We can imagine a fluorescent series $\stackrel{\sim }{x}(t)$, 90 degrees out of phase with the original measurements $x(t)$. This replica is known as the Hilbert Transform^30^ and can be computed under very general conditions for a periodic process^31^. Viewing the doublet ($x\left(t\right),\stackrel{\sim }{x}(t))$ living on the complex plane, the Hilbert Phase ${F}^{H}\left(t\right)$ is defined as the angle between $x(t)$ and $\stackrel{\sim }{x}\left(t\right){:}$

$${F}^{H}\left(t\right)= {tan}^{-1}\frac{\stackrel{\sim }{x}(t)}{x\left(t\right)}$$

The Hilbert phase is continuized because of discontinuities at − $\pi$ and $\pi$ in ${tan}^{-1}$ and denoted by ${F}^{C}\left(t\right)$^31^. In that the experiments here were designed to synchronize the cellular oscillators at time 0 by placing all cells in the light for 26 h initially, the Hilbert Phase is shifted to pass through the origin in Fig. 6 and divided by 2 $\pi$ to measure phase ${M}^{C}({t}_{1},{t}_{0}$) in cycles completed from time ${t}_{0}$ to time ${t}_{1}$:

$${M}^{C}\left({t}_{1},{t}_{0}\right)= \frac{{F}^{C}\left({t}_{1}\right)-{F}^{C}\left({t}_{0}\right)}{2\pi }$$

This phase measure increases linearly with time for a sinusoidal process, but for a process experiencing synchronization the phase curve is nonlinear^5^—the phases of cellular clocks change towards each other as they synchronize.

### The ensemble for the clock stochastic network is determined by parallel tempering

In order to escape local optima in (2) searching for the ensemble distribution, K replicas of the original system in (2) are created on the computer in parallel tempering. Each replica has its own temperature in (2). Those replicas with a higher temperature have a flatter surface in (2) as a function of the parameters $\Theta$. Those replicas with a lower temperature have a more peaked surface as function of the parameters. The higher temperature replicas are free to explore more of the parameter space than lower temperature replicas. Each replica is allowed to engage in its own Markov Chain Monte Carlo (MCMC) search for good $\Theta$’s as defined by (2) using previous defined methods^18^ called in-chain iterations, but also each replica communicates its $\Theta$-vector with swaps with the neighboring temperature. Higher temperature replicas find promising solutions, which are then communicated to lower temperature replicas. The combination of MCMC in-chain search by replicas with swaps between replicas allows the escape of local optima^14^. The lowest temperature replica then yields the final solution to the problem and is called the target replica. It has been our experience that more standard MCMC methods, such as Metropolis–Hastings, were not sufficient for the identification of stochastic networks^14^.

To setup a parallel tempering run a temperature grid ${T}_{1}<{T}_{2}<\dots <{T}_{K}$ is constructed with ${T}_{1}=1$ corresponding to our target replica (our solution to the problem). Swaps are defined between temperatures ${T}_{i}$ and ${T}_{j}$ by an acceptance probability:

$${\rho }_{ij}=min\left\{1,\frac{{\mathbb{Q}}_{{T}_{i}}({x}_{\left(j\right)}){\mathbb{Q}}_{{T}_{j}({x}_{\left(i\right)})}}{{\mathbb{Q}}_{{T}_{i}}({x}_{\left(i\right)}{){\mathbb{Q}}}_{{T}_{j}({x}_{\left(j\right)})}}\right\}$$

The vector ${x}_{\left(i\right)}$ are the parameters of the ith replica. As swaps are made, interesting solutions at high temperatures can move to colder temperatures and ultimately may be incorporated into the target solution at ${T}_{1}=1$. Solutions move through the temperature grid, and an effective temperature grid has substantial communication between neighbors. No temperatures become isolated.

### Choosing the grid (K) and temperatures in parallel tempering.

The temperature grid ${T}_{1}<{T}_{2}<\dots <{T}_{K}$ was chosen as described previously^70^. The number of replicas was initialized by K $=\sqrt{D}$, where D = 35 is the number of parameters in $\theta$. Using the initial guess ${\theta }^{0}$ for the parameters, the largest temperature was set to ${T}_{K}=\frac{{\chi }^{2}\left({\theta }^{0}\right)}{30}$.

The in-chain updates (i.e., Metropolis–Hastings updates) were run for 200 iterations^14^. If the acceptance rate for in-chain updates fell within (0.6, 0.75), the temperature was accepted; otherwise, the temperature ${T}_{K}$ was changed. Then another 200 iterations were tried. If the acceptance rate for in chain updates fell within (0.6, 0.75), then the new temperature was accepted; otherwise, the cycle was repeated until the trial temperature fell within (0.6, 0.75). In this way the goal is to add temperatures so that the exchange rate between temperatures remains constant and nonzero.

A linear grid with K temperatures was established by the following protocol using a target swap rate of 0.4 for neighboring replicas:

1. perform an update of parameters $\theta$ for each replica.
2. propose swaps between neighboring replicas 1 and 2, 3 and 4, 5 and 6,…
3. also propose swaps between neighboring replicas 2 and 3, 4 and 5, 6 and 7,…
4. repeat steps 1), 2) and 3), 200 times.

For each pair of neighboring replicas (*i,* *i* + 1) the following quantity was calculated:

$${\mathbb{Q}}_{i,i+1}=\frac{1}{{N}_{swap}^{i,i+1}}\sum_{l=1}^{{N}_{swap}^{(i,i+1)}}ln\left({\rho }_{i,i+1}^{l}\right),$$

The quantity ${N}_{swap}^{\left(i,i+1\right)}$ is the number of proposed swaps between replicas *i* and *i* + 1, and ${\rho }_{i,i+1}^{l}$ is the acceptance probability of the *lt*ℎ proposal for swapping *i* and *i* + 1.

If ${R}_{i,i+1}=\left[\sqrt{\frac{{\mathbb{Q}}_{i,i+1}}{\mathrm{ln}(0.4)}}\right]>0$, then a grid ${R}_{i,i+1}$ temperature is added , evenly spaced between ${T}_{i}$ and ${T}_{i+1}$_._

This temperature grid creation process was performed 3 times to make sure there were enough temperatures on the grid to prevent isolation of replicas on the grid.

Temperatures between ${T}_{1}$ and ${T}_{k}$ were added as follows.

1. Perform parallel tempering using the above steps 1), 2) and 3) 350 times. with the new temperature set .
2. For each temperature *T*_*i*_ calculate the flow fraction
  $$f\left({T}_{i}\right)=\frac{{n}_{up}\left({T}_{i}\right)}{{n}_{up}\left({T}_{i}\right)+{n}_{down}\left({T}_{i}\right)},$$

where ${n}_{up}\left({T}_{i}\right)$ and ${n}_{down}\left({T}_{i}\right)$ are the total number of replicas that were drifting upward and downward, respectively when they visited *T*_*i*_.

3. Linearly interpolate *f* between temperatures.
4. Calculate the inverse function *g* of *f.*
5. Change the temperature values from *T*_*i*_ to ${T}_{i}^{new}=g\left(1- \frac{i-1}{K-1}\right)$.

This process of shifting the intermediate temperatures was repeated 3 times.

The shifting of temperatures was done to optimize the flow of replicas through the temperature grid so that no temperature becomes isolated and unable to swap with other temperatures.

After the steps above to choose the temperature grid, the parallel tempering algorithm was performed for about 30,000 Monte Carlo updates in Fig. 3A, where by update we mean the steps 1), 2) and 3) described in the add-temperature process. In implementing this temperature grid above, three initial conditions for the parameters were tried, and in one of the MCMC runs the target replica at temperature ${T}_{1}$ stopped swapping with the neighboring temperature late in equilibration. To eliminate this problem the linear temperature grid was allowed to increase again to include 60 temperatures during equilibration in Fig. 3A.

### The ensemble for the clock stochastic network is determined by genetic algorithms supplemented by Metropolis–Hastings Monte Carlo

Two genetic algorithms were employed here^28,29^. The two algorithms used in the simulations are part of the family of genetic algorithms known as Particle Swarm Optimization (PSO) algorithms. PSO algorithms try to optimize a function **f** defined on a domain $\mathcal{D}$ by dividing a population of particles **x**_**i**_ in $\mathcal{D}$**,** i = 1,2,…,sz, into groups called swarms and letting these swarms look for regions in the parameter space that could contain the optimum value of **f**. To make the exploration of the parameter space more effective, the swarms are encouraged to share information among themselves. Usually, 90% of the total number of generations are used to explore the parameter space to find promising region(s) that could contain optimum values of **f**. The exploration phase is followed by exploitation, whereby the algorithm speeds up the convergence of particles to an optimum value of f. In the following, we assume that we want to minimize the function f, *e.g*., the ${\chi }^{2},$ in Eq. (2), so f(x_1_) < f(x_2_) means x_1_ is better than x_2_.

The Dynamic Multi-Swarm Particle Swarm Optimizer with Cooperative Learning Strategy (DMS-PSO-CLS) genetic algorithm is now briefly described^29^. It has three features for optimization: (1) swarms of particles, moving in the parameter space with the best particle of a swarm being denoted by *pbest*; (2) a culling/recombination stage at the end of a generation where each parameter of the two worst particles in each swarm is replaced by the corresponding parameter of one of the pbest particles; (3) a regrouping between particles (or migration between swarms) every RR generations Then the process is repeated in each succeeding generation, 600 to 1,000 generations.

### Swarm movement

Each of sz particles with NN = 4 particles per swarm in MM swarms has an inertia of *w*, which decreases linearly with generation, the initial value being ${w}_{1}$ and the final value being ${w}_{2}.$ The acceleration constants ${c}_{1}$ and ${c}_{2}$ determine in part the genetic algorithms in Table 1. Each particle $i$ has a position component ${x}_{i}^{d}$ and velocity component ${v}_{i}^{d}$ on parameter d in the D-dimensional parameter space, d = 1,2,…,D. The dimension D is 35 here. All parameters in the model are rescaled to the unit cube ${[\mathrm{0,1}]}^{35}.$ Initial conditions were chosen as part of a Sobol space-filling sequence in the parameter space^71^ or randomly from within the unit cube^40^. Equations of motion of the swarm are given by^29^:

3 $$\begin{gathered} v_{i}^{d} \leftarrow wv_{i}^{d} + c_{1} \cdot rand1_{i}^{d} \left( {pbest_{i}^{d} - x_{i}^{d} } \right) + c_{2} \cdot rand2_{i}^{d} \left( {lbest_{i}^{d} - x_{i}^{d} } \right) \hfill \\ x_{i}^{d} \leftarrow x_{i}^{d} + v_{i}^{d} \hfill \\ \end{gathered}$$

for the exploration phase and by

4 $$\begin{gathered} v_{i}^{d} \leftarrow wv_{i}^{d} + c_{1} \cdot rand1_{i}^{d} \left( {pbest_{i}^{d} - x_{i}^{d} } \right) + c_{2} \cdot rand2_{i}^{d} \left( {gbest^{d} - x_{i}^{d} } \right) \hfill \\ x_{i}^{d} \leftarrow x_{i}^{d} + v_{i}^{d} \hfill \\ \end{gathered}$$

For the exploitation phase*.*

The vector ${{\varvec{p}}{\varvec{b}}{\varvec{e}}{\varvec{s}}{\varvec{t}}}_{{\varvec{i}}}$ is a particle’s historically best position in the parameter space according to (2). The vector ${{\varvec{l}}{\varvec{b}}{\varvec{e}}{\varvec{s}}{\varvec{t}}}_{{\varvec{i}}}$ is the historically best position in an ith particle’s swarm. The vector gbest represents the position of the globally best solution, i.e., the best particle in the whole population. Note that when lbest and gbest are calculated, the historically best solution of all particles in a swarm and historically best of particles in the whole population are being recorded, respectively. The quantities ${rand1}_{i}^{d}$ and ${rand2}_{i}^{d}$ are uniform random numbers drawn from [0,1] that vary with each update to the velocity of a particle.

### Culling and recombination

The genetic algorithm in row 2 (Table 1) with ${\chi }^{2}=2773.07$ is used to illustrate culling and recombination. For each dimension d, there is a random draw of size 2 from the 80/4 = 20 pbest particles moving on the parameter space. For each dimension d of the parameter space, 2 of the pbest particles are randomly chosen, and the best one of them will donate parameter d to one of the worst particles. The method is repeated for second worst particles. Since this random draw of 2 best particles is redone for each dimension, it is possible that the 2 worst particles will have contributions from more than 2 of the best particles. In other words, there is recombination between all of the best particles in culling the 2 worst particles from each swarm.

### Regrouping (or migration)

Particles are randomly regrouped every RR generations. The constants used were: (1) ${w}_{1}$= 0.9; (2) ${w}_{2}$= 0.4; (3) ${c}_{1}=$ 1.49445; (4) ${c}_{2}$ = 1.49445; (5) ${v}_{max}$ = 0.2; (6) ${v}_{min}$ = − 0.2 (7) RR = 5^29^; (8) ${w}_{3}=0.2$^29^; (9) T = 1 in Eq. (2). Inertia weight *w* was decreased linearly from *w*_*1*_ to *w*_*2*_ during exploration phase and was kept constant at ${w}_{3}$ during the exploitation phase. See^29^.

An alternative genetic algorithm named Particle Swarm Optimization with Dynamic Learning Strategy (PSO-DLS)^28^ was also tried (Table 1). There were only two stages, swarm movement and migration. With probability 1-p, a particle does not communicate with other swarms, and its movement is described by (3). With probability p a particle does communicate with other swarms, and its movement is described by (5):

5 $$\begin{gathered} v_{i}^{d} \leftarrow wv_{i}^{d} + c_{1} \cdot rand1_{i}^{d} \left( {pbest_{i}^{d} - x_{i}^{d} } \right) + c_{2} \cdot rand2_{i}^{d} \left( {\frac{1}{MM}\mathop \sum \limits_{m = 1}^{MM} lbest_{m}^{d} - x_{i}^{d} } \right) \hfill \\ x_{i}^{d} \leftarrow x_{i}^{d} + v_{i}^{d} \hfill \\ \end{gathered}$$

Equation (4) is used during the exploitation phase*.*

This is sometimes referred to as the admixture model of migration with p as the migration rate^72^. The same acceleration constants, ${c}_{1}$ and ${c}_{2}$, were used and set to 1.49445. This admixture parameter linearly increases with generation t according to p = t/iter, where iter is the total number of exploration generations (in our case 540 or 900 in Table 1). There is pseudocode available^28^.

Metropolis–Hastings accumulation followed equilibration using the 12 best solutions in Table 1 and were combined at the end to produce a reconstruction of the likelihood in (2). A total of 14,000 total updates were performed for each of the 12 chains. The first 3,500 updates were used to adjust the parameter step widths in the Metropolis–Hastings algorithm and were discarded^73^. From the remaining 10,500 updates, every 35th model was sampled for a total of 300 samples from each of the 12 chains. The final sample for the accumulation run consisted of 12 × 300 = 3,600 models. Summary statistics on each model parameter for the fitted ensemble are given in Table 1.

## Supplementary information

Supplementary file1

Supplementary file2
