# Supplementary material for: Identifying a stochastic clock network with light entrainment for single cells of Neurosporacrassa
Source: Sci Rep. 2020 Sep 16;10:15168. doi: 10.1038/s41598-020-72213-1 (PMC7495483; doi:10.1038/s41598-020-72213-1)
Supplement: Supplementary file 1 — Supplementary file1 [file 41598_2020_72213_MOESM1_ESM.docx]

**SUPPLEMENTARY MATERIALS FOR**

Identifying a stochastic clock network with light entrainment for single cells of *Neurospora crassa*

by

**Authors:** Cristian Caranica, Ahmad Al-Omari , Heinz-Bernd Schüttler, and Jonathan Arnold

**Corresponding authors**: Jonathan Arnold, [arnold@uga.edu](mailto:arnold@uga.edu)

**This PDF includes:**

Table S1

Table S2

Fig.s S0, S1, S2, S3, S4, S5, S6 and caption to video S1

**Other supplementary materials for this manuscript include:**

Video

**Table S1**. Best parameters from accumulation run generated from 12 equilibration runs using genetic algorithms. This best model in the accumulation run had a $\chi^{2}$ = 2671.95.

| U_r0 | 245 |
| --- | --- |
| u_r1 | 298 |
| u_p | 249 |
| f_0 | 0 |
| f_1 | 1 |
| f_r | 344 |
| f_p | 239 |
| w | 148 |
| g_0 | 0 |
| g_1 | 1 |
| g_r | 156 |
| g_p | 366 |
| A | 0 |
| abar | 0.2965 |
| S1 | 20.1747 |
| S3 | 0.0874 |
| S4 | 5.5247 |
| D1 | 6.1832 |
| D3 | 0.116 |
| C1 | 0.0001 |
| L1 | 2.1762 |
| L3 | 0.0014 |
| D4 | 0.4193 |
| D6 | 27.2571 |
| D7 | 0.0066 |
| D8 | 0.0008 |
| C2 | 499.928 |
| P | 0 |
| Ac | 0 |
| Bc | 0.005 |
| Sc | 9.1158 |
| Lc | 0.0018 |
| Dcr | 0.0662 |
| Dcp | 0.0224 |
| fIl | 22.4643 |

**Table S2**. Only the initial molecular counts are varied with the alteration in the mRNA/DNA and protein/DNA ratios as shown below. As a consequence there is no change in the dynamics of the system when the “size of the cell” is varied through the relation in Fig. 5.

| Model parameters | Ratios are X/7 | Ratios are 1 | Ratios are X15 |
| --- | --- | --- | --- |
| U_r0 | 245/7 | 245 | 245X15 |
| u_r1 | 298/7 | 298 | 298X15 |
| u_p | 249/7 | 249 | 249X15 |
| f_0 | 0/7 | 0 | 0X15 |
| f_1 | 1/7 | 1 | 1X15 |
| f_r | 344/7 | 344 | 344X15 |
| f_p | 239/7 | 239 | 239X15 |
| w | 148/7 | 148 | 148X15 |
| g_0 | 0/7 | 0 | 0X15 |
| g_1 | 1/7 | 1 | 1XX15 |
| g_r | 156/7 | 156 | 156X15 |
| g_p | 366/7 | 366 | 366X15 |
| A | 0 | 0 | 0 |
| abar | 0.2965 | 0.2965 | 0.2965 |
| S1 | 20.1747 | 20.1747 | 20.1747 |
| S3 | 0.0874 | 0.0874 | 0.0874 |
| S4 | 5.5247 | 5.5247 | 5.5247 |
| D1 | 6.1832 | 6.1832 | 6.1832 |
| D3 | 0.116 | 0.116 | 0.116 |
| C1 | 0.0001 | 0.0001 | 0.0001 |
| L1 | 2.1762 | 2.1762 | 2.1762 |
| L3 | 0.0014 | 0.0014 | 0.0014 |
| D4 | 0.4193 | 0.4193 | 0.4193 |
| D6 | 27.2571 | 27.2571 | 27.2571 |
| D7 | 0.0066 | 0.0066 | 0.0066 |
| D8 | 0.0008 | 0.0008 | 0.0008 |
| C2 | 499.928 | 499.928 | 499.928 |
| P | 0 | 0 | 0 |
| Ac | 0 | 0 | 0 |
| Bc | 0.005 | 0.005 | 0.005 |
| Sc | 9.1158 | 9.1158 | 9.1158 |
| Lc | 0.0018 | 0.0018 | 0.0018 |
| Dcr | 0.0662 | 0.0662 | 0.0662 |
| Dcp | 0.0224 | 0.0224 | 0.0224 |
| fIl | 22.4643 | 22.4643 | 22.4643 |


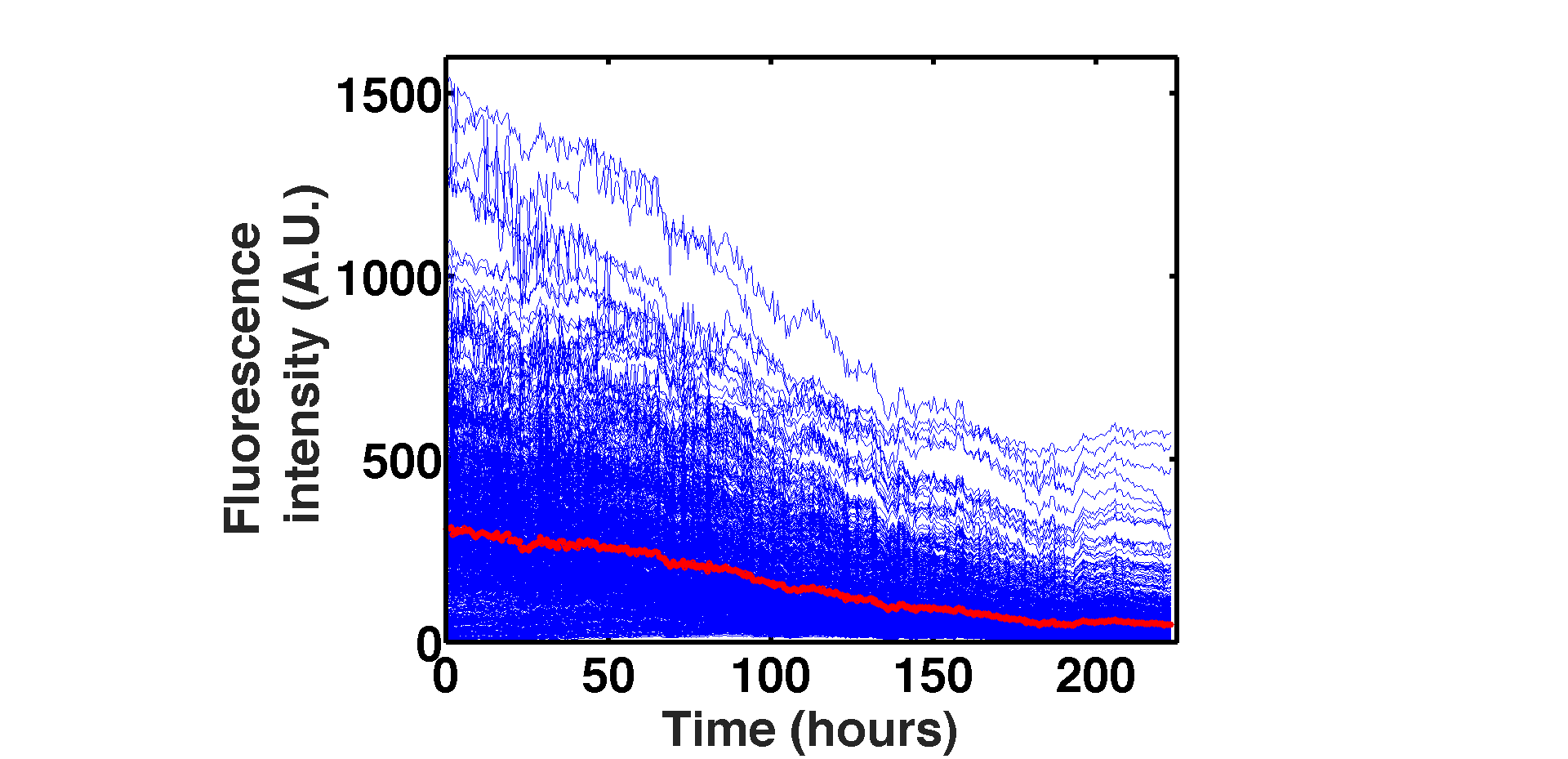


**Fig S0**. Stochastic variation in 868 Single Cell Trajectories for MFNC9 strain over ten days with measurements every half hour. The blue curves are single cell trajectories in isolated droplets. The red curve is the average trajectory of the 868 cells. Figure is from earlier work^1^.

This fit to the average periodograms of the single cell data was unsatisfactory as shown in Fig 3. While the data on the D/D experiment were quite well fitted by the model ensemble under the neutral model in Fig 1,

**Fig S1. The predicted periodograms of the neutral model with no intercell communication was fitted with parallel tempering to the observed periodograms of 4 experiments, one D/D/ and three L/D with 6 h, 12 h, and 36 h days, respectively, with two major discrepancies for the 6 h day and 12 h days each with its two peaks. Each periodogram represents an average over the individual periodograms of at least 1,000 single cells. The model appeared only to fit one of the peaks of the single cell data in the 6 h day. The fitted periodograms were obtained by an accumulation run with updates from a particular kind of MCMC method called parallel tempering (see Materials and Methods). Observations were taken at half hour intervals over L equidistant observation times. The duration of the experiment is T. The sampled frequencies in the periodogram are denoted by** $\boldsymbol{f}_{\boldsymbol{l}}\boldsymbol{=}\frac{\boldsymbol{l}}{\boldsymbol{T}}\boldsymbol{, l=1,\ldots}\left[ \frac{\boldsymbol{L}}{\boldsymbol{2}} \right]\boldsymbol{.}$ **The first 240 indices** $\boldsymbol{l of}$ **frequencies in the periodogram are for the D/D experiment. The next 256 indices of frequencies are for the L/D experiment with a 6 h day. The next 201 indices are for the L/D with a 12 h day. The last 256 indices are for a 36 h day. For the D/D experiment the x-axis is the index** $\boldsymbol{l}$**. The x-axis is**$\boldsymbol{l}$ **with a shift of 240 for a 6 h day, then with 240+256 for a 12 h day, and finally with 240+256+201 for a 36 h day to separate out the periodograms on the same graph. The periodograms of the experiments and the model were Rhodamine B normalized, detrended, and bias-corrected as described in materials and methods. The plot was created in MATLAB_R2018B (**<https://www.mathworks.com/products/matlab.html>).

the model ensemble did not track well to the data for the 6 h and 12 h day. In both cases there were two peaks in the power spectrum, but the 6 h and 12 peaks in Fig 3 were not well predicted by the model. One possibility is that the model ensemble failed to capture fully the entrainment to light in Fig 3 because the illumination parameter $f_{IL}$ was fixed at 2. During the third stage of the equilibration process, the illumination parameter was allowed to float, but did not depart from 2 in Fig 2A during equilibration. Allowing an extra degree of freedom and more equilibration steps, however, did improve the fit slightly (Fig 2A).

There are two further possible explanations for the lack of fit in Fig 3. One, parallel tempering is failing to find the best models by optimizing Equation (2) or two, there are two populations of oscillators for the 6 h and 12 h day, indicating a limitation on light entrainment. To test these hypotheses we implemented new fitting algorithms called genetic algorithms (see Materials and Methods) to maximize Equation (2) during the equilibration phase of Markov Chain Monte Carlo followed up by Metropolis-Hastings Monte Carlo in the accumulation phase^2^.


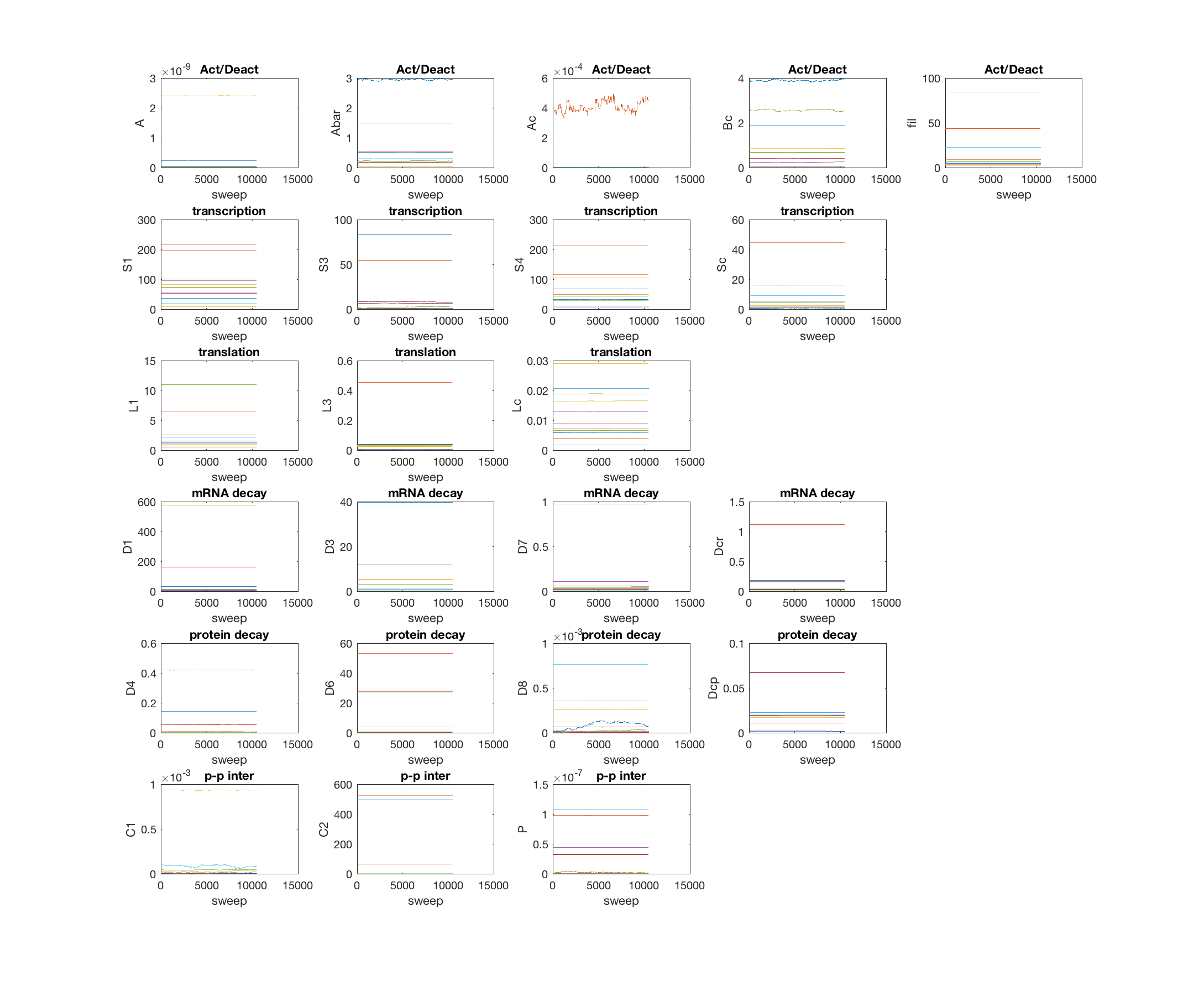


**Fig S2**

Plots of rate constants vs. sweeps in the combined accumulation run (Table 1) supported the ensemble was equilibrated. Each plot has twelve curves from 12 distinct MCMC accumulation runs initiated with 12 distinct genetic algorithms. All plots are without trend as expected for an equilibrated MCMC run. The rows of plots are for rates of: (1) gene activation and deactivation; (2) transcription; (3) translation; (4) mRNA decay; (5) protein decay; (6) protein-protein interactions (p-p inter). In a particular plot there may appear to be fewer than 12 curves when the curves overlap. The plots were created in MATLAB_R2018B **(**<https://www.mathworks.com/products/matlab.html>).


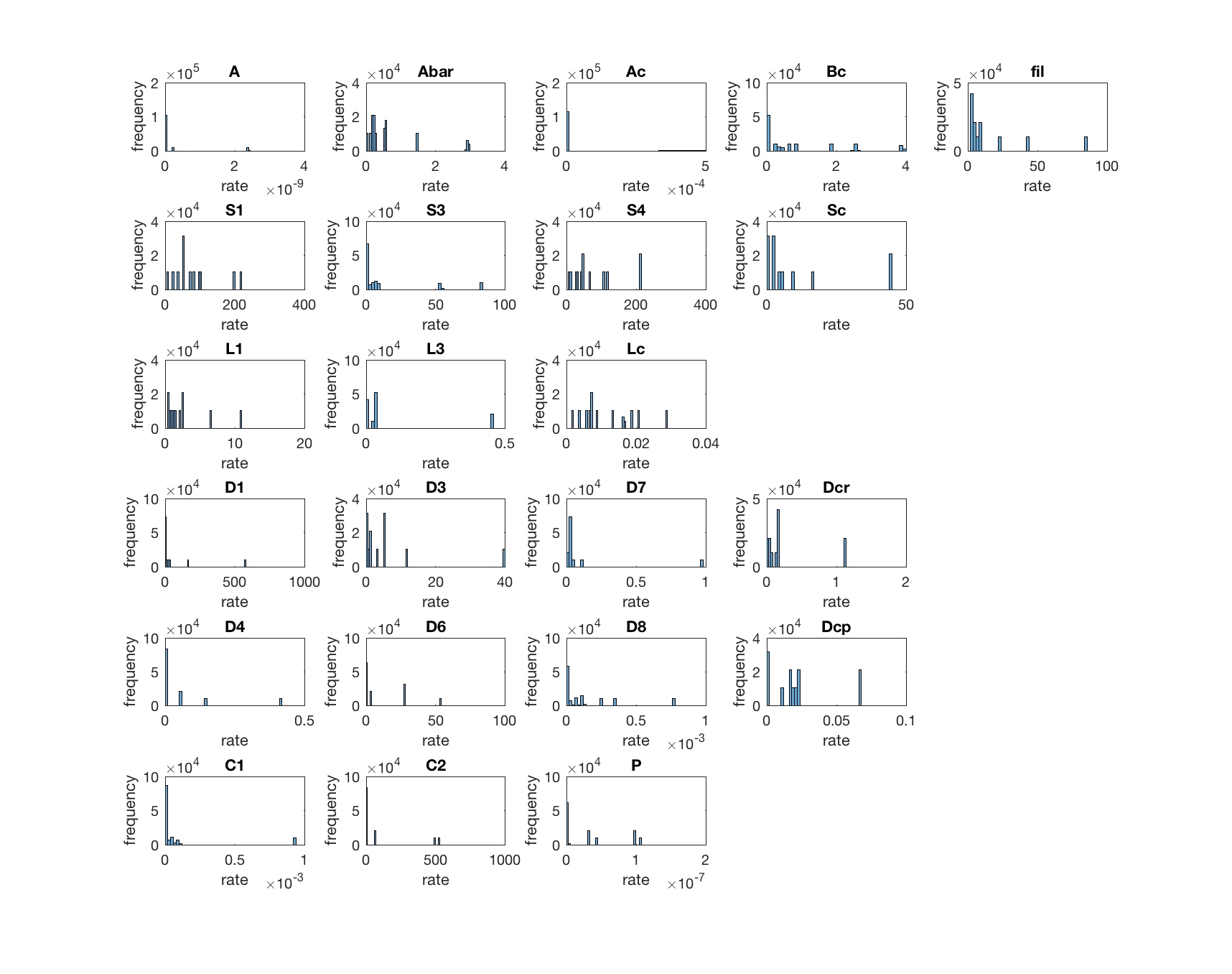


**Fig S3**

Histograms of 126,000 parameter values for each of the 23 rate constants, showing the variation across the model ensemble (Table 2) and due to stochastic intracellular variation. The rows of histograms are for rates of: (1) gene activation and deactivation; (2) transcription; (3) translation; (4) mRNA decay; (5) protein decay; (6) protein-protein interactions. The plots were created in MATLAB_R2018B **(**<https://www.mathworks.com/products/matlab.html>).


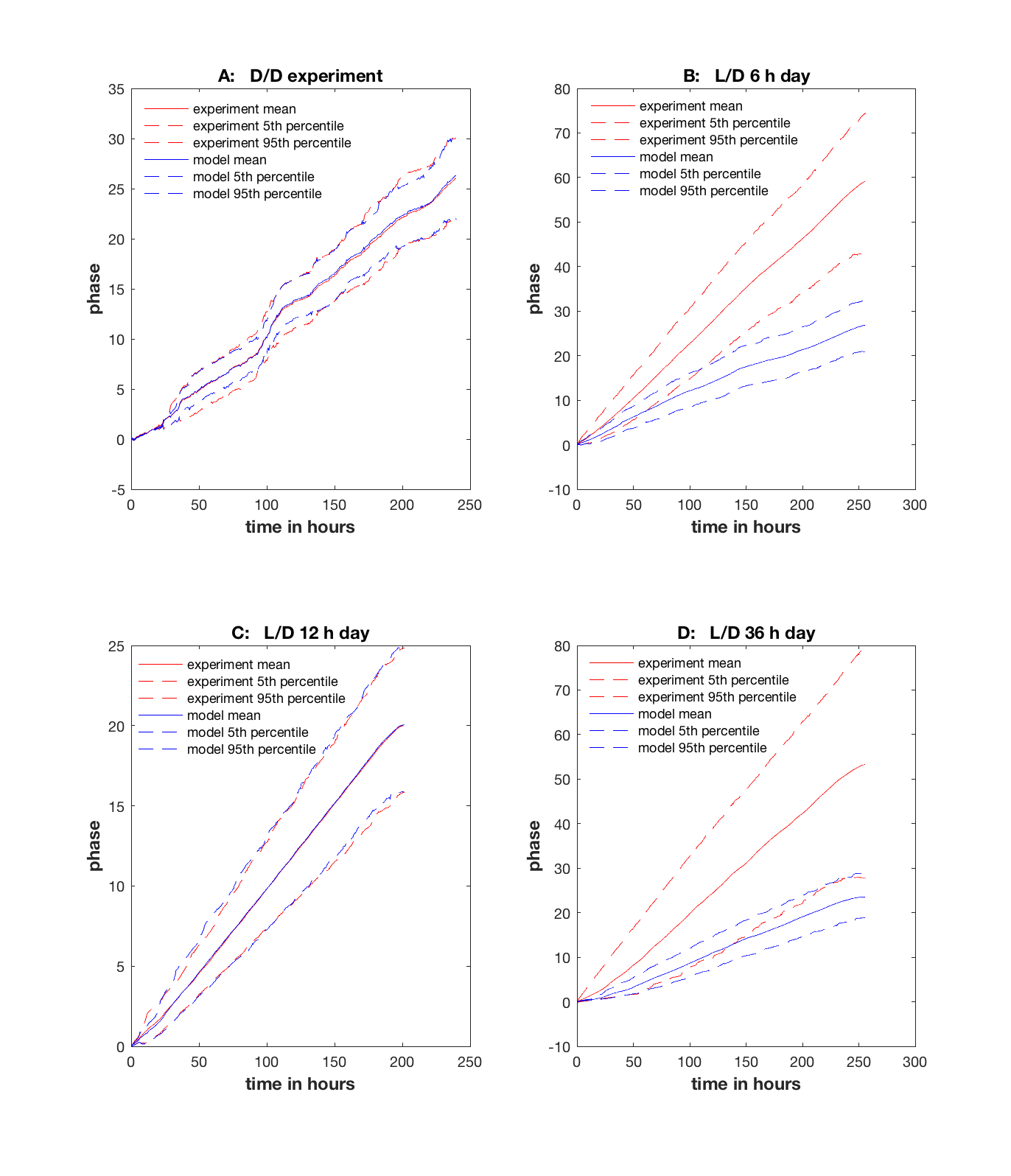


**Fig S4 .**

The phase plots as a function of time indicated that there are limitations on goodness of fit for the 6 h day L/D experiment and possibly the 18 h L/D exxperiment. The 95^th^ percentile, the mean, and the 5^th^ percentile of the phase for all cells are graphed for each experiment (red) and the model (blue) computed from 1,024 Gillespie Trajectories from a fitted ensemble with RNA/DNA ratio and protein/DNA ratio of 64 and 300, respectively. The change in noise was done in such a way to preserve the rate constants (see Materials and Methods). Single cell trajectories for data and model are summarized under the: (A) D/D experiment; (B) 6 h day L/D experiment; 12 h day L/D experiment; (D) 36 h day L/D experiment. The plots were created in MATLAB_R2018B **(**<https://www.mathworks.com/products/matlab.html>).

**
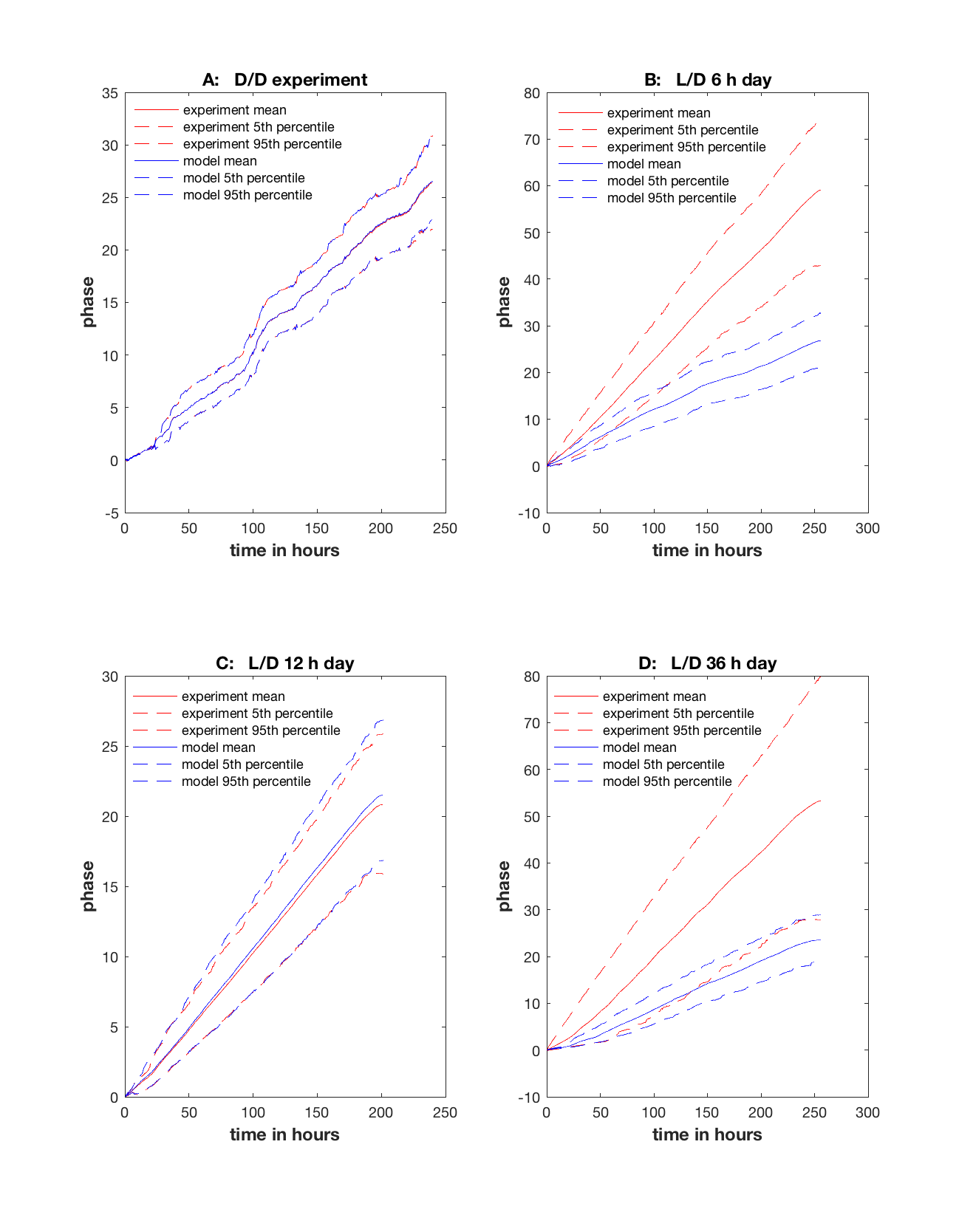
 Fig S5.**

The phase plots as a function of time indicated that there are limitations on goodness of fit for the 6 h day L/D experiment and possibly the 18 h day L/D experiment. The 95^th^ percentile, the mean, and the 5^th^ percentile of the phase for all cells are graphed for each experiment (red) and the model (blue) computed from 1,024 Gillespie Trajectories from a fitted ensemble with RNA/DNA ratio and protein/DNA ratio of 100 and 380, respectively. The change in noise was done in such a way to preserve the rate constants (see Materials and Methods). Single cell trajectories for data and model are summarized under the: (A) D/D experiment; (B) 6 h day L/D experiment; 12 h day L/D experiment; (D) 36 h day L/D experiment. The plots were created in MATLAB_R2018B **(**<https://www.mathworks.com/products/matlab.html>).


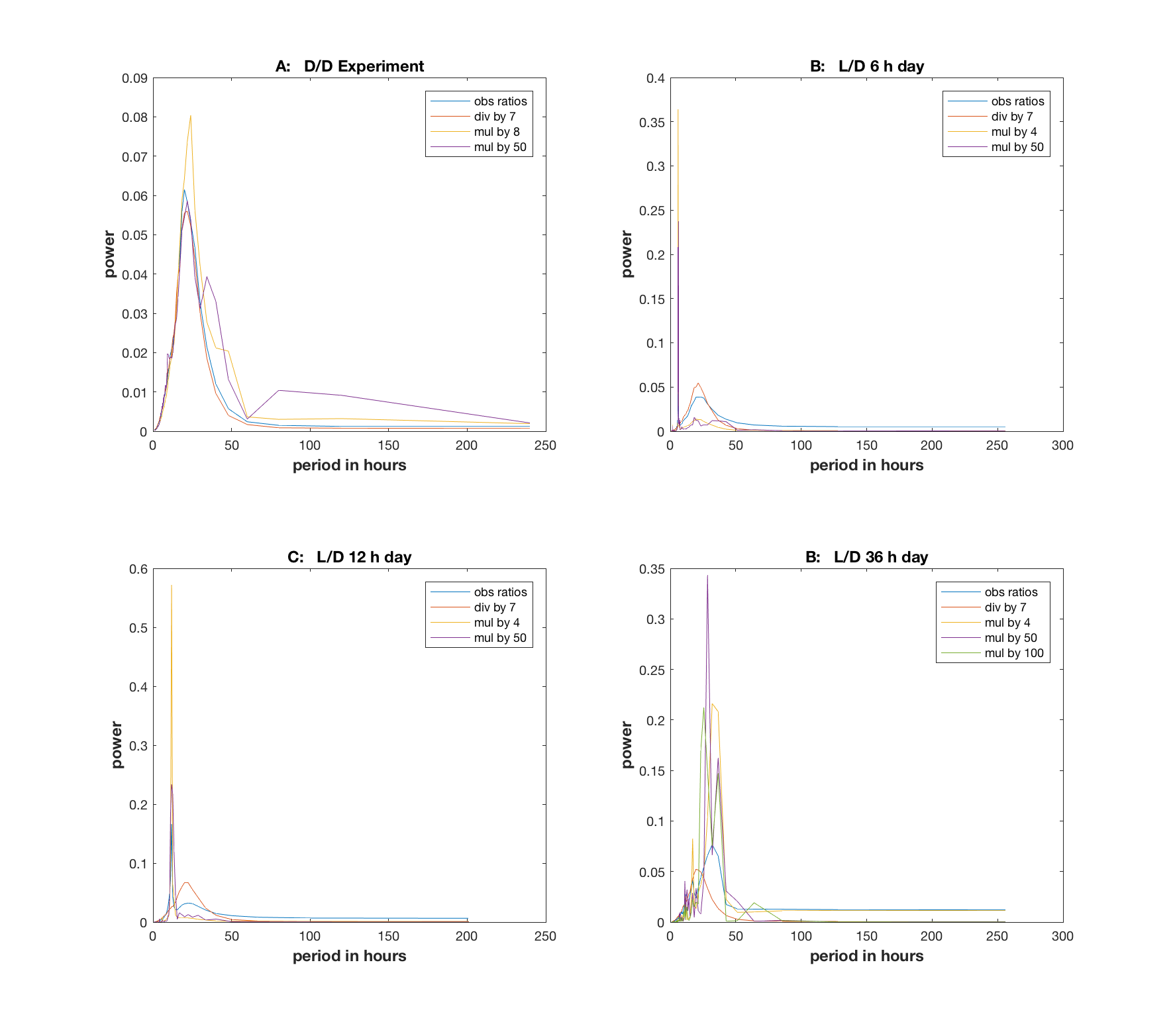


**Fig** S6

The power at the driven frequency or intrinsic frequency of a cellular oscillator is a nonlinear function of the stochastic intracellular noise. The stochastic intracellular noise was varied by multiplying/dividing the RNA/DNA and protein/DNA ratios in such a way as not to change the rate constants. (A) D/D experiment; (B) L/D 6 h day; (C) L/D 12 h day; (D)L/D 36 h day. The periodograms are normalized so that the area under each periodogram is 1. The model used to generate the periodograms above is the best fitting model in S1 Table. The plots were created in MATLAB_R2018B **(**<https://www.mathworks.com/products/matlab.html>).

.

**Legend to video.**

A total of 1,000 Gillespie trajectories of a 1,000 cells with a driving light signal from a 12 hour day and with their stochastic intracellular noise are overlayed one by one to reinforce the oscillatory signal at the stochastic resonance. The individual Gillespie trajectories appear highly synchronized.

References

1 Deng, Z. *et al.* Synchronizing stochastic circadian oscillators in single cells of *Neurospora crassa*. *Scientific Reports* **6**, 35828 (2016).

2 Caranica, C. *et al.* Ensemble methods for stochastic networks with special reference to the biological clock of *Neurospora crassa*. *PloS one* **13**, e0196435 (2018).
